# Causal effects of systemic inflammatory regulators on chronic kidney diseases and renal function: a bidirectional Mendelian randomization study

**DOI:** 10.3389/fimmu.2023.1229636

**Published:** 2023-08-30

**Authors:** Hongdian Li, Mingxuan Li, Cong Liu, Pengfei He, Ao Dong, Shaoning Dong, Mianzhi Zhang

**Affiliations:** ^1^ Department of Nephrology, Dongfang Hospital, Beijing University of Chinese Medicine, Beijing, China; ^2^ Department of Cardiology, Beijing Hospital of Traditional Chinese Medicine, Capital Medical University, Beijing, China; ^3^ Department of Nephrology, Tianjin Academy of Traditional Chinese Medicine Affiliated Hospital, Tianjin, China

**Keywords:** chronic kidney disease, systemic inflammation, inflammatory modulators, Mendelian randomization, genetic causal association

## Abstract

**Background:**

While targeted systemic inflammatory modulators show promise in preventing chronic kidney disease (CKD) progression, the causal link between specific inflammatory factors and CKD remains uncertain.

**Methods:**

Using a genome-wide association study of 41 serum cytokines from 8,293 Finnish individuals, we conducted a bidirectional two-sample Mendelian randomization (MR) analysis. In addition, we genetically predicted causal associations between inflammatory factors and 5 phenotypes, including CKD, estimated glomerular filtration rate (eGFR), dialysis, rapid progression of CKD, and rapid decline in eGFR. Inverse variance weighting (IVW) served as the primary MR method, while MR-Egger, weighted median, and MR-pleiotropy residual sum and outlier (MR-PRESSO) were utilized for sensitivity analysis. Cochrane’s Q test for heterogeneity. Leave-one-out method ensured stability of MR results, and Bonferroni correction assessed causal relationship strength.

**Results:**

Seventeen cytokines were associated with diverse renal outcomes. Among them, after Bonferroni correction test, higher tumor necrosis factor alpha levels were associated with a rapid decrease in eGFR (OR = 1.064, 95% CI 1.028 – 1.103, *P* = 0.001), higher interleukin-4 levels were associated with an increase in eGFR (β = 0.003, 95% CI 0.001 – 0.005, *P* = 0.002), and higher growth regulated oncogene alpha (GROα) levels were associated with an increased risk of CKD (OR=1.035, 95% CI 1.012 - 1.058, *P* = 0.003). In contrast, genetic susceptibility to CKD was associated with an increase in GROa, and a decrease in eGFR may lead to an increase in stem cell factor. We did not find the presence of horizontal pleiotropy during the analysis.

**Conclusion:**

We discovered causally related inflammatory factors that contribute to the initiation and progression of CKD at the genetic prediction level.

## 1 Introduction

Chronic kidney disease (CKD) is a growing global health burden with increasing prevalence and incidence. According to a systematic analysis of the Global Burden of Disease Study, the global mortality rate from CKD has increased by 41.5% between 1990 and 2017, resulting in approximately 1.23 million deaths in 2017 (1). Moreover, CKD imposes a substantial economic burden, with the cost of CKD and ESRD representing 24% of total annual Medicare expenditures in the United States (2). In low- and middle-income countries, more than half of ESDR patients are unable to continue dialysis due to the high cost of treatment (3–5). Despite the high prevalence and economic burden of CKD, there is a lack of effective treatments to slow the progressive loss of renal function and the development of ESRD (6). Therefore, a better understanding of the pathogenesis of CKD is essential to identify new treatment options.

Inflammation may be a promising target for intervention in CKD (7). Regardless of the etiology of CKD, chronic inflammation may be present as a cause and consequence of glomerular and tubulointerstitial pathology (8–11), as a microinflammatory state significantly different from that of the normal renal function population is observed in patients with many forms of CKD with asymptomatic proteinuria (12, 13). Although there are several mechanisms that contribute to the pathological alterations of glomeruli and tubules, inflammation is the key link between them. Transcription factors induce chronic hypoxia in the tubular mesenchyme, leading to peripheral capillary sparing, which triggers adverse phenotypes such as apoptosis. Consequently, mediators mediate inflammatory cell infiltration and fibrosis, impairing local oxygenation and causing aseptic inflammation (14–16). Persistent microinflammation aggravates reactive oxygen species (ROS) loss, exacerbating progressive renal function decline in a mutually reinforcing manner throughout CKD progression (17–19). Oxidative stress induces inflammation through the activation of nuclear factor kappa-B (NF-κB) (20), and the subsequent production of inflammatory factors is associated with a progressive decrease in estimated glomerular filtration rate (eGFR) (21, 22). Observational studies have shown that inflammation is one of the most important pathways for the decline in renal function in European patients with nine different types of CKD (23). These evidences suggest that inflammation is directly associated with CKD and its complications and that inflammation is both the initiator and the outcome of a vicious cycle. Current population-based clinical studies have not yet demonstrated a direct causal association between inflammation and CKD.

Mendelian randomization (MR) uses genetic variants to establish causal links, overcoming biases, resembling nature’s own large-scale randomized controlled trial (RCT) (24). A recent MR analysis has shed new light on the potential therapeutic role of inflammatory modulators in CKD by identifying a causal association between elevated C-reactive protein levels and diabetic nephropathy (25). Bidirectional MR analysis, an extension of conventional MR, has been instrumental in untangling intricate relationships in biological systems, including feedback loops between exposure and outcome variables (26).

To comprehensively evaluate the causal association between systemic inflammatory regulators and CKD, we conducted a bidirectional MR study. Given the prolonged progression of CKD, we included multiple endpoints in our analysis, including eGFR, rapid decline in renal function (Rapid3, defined as a decline in eGFR exceeding 3 mL/min/1.73 m^2^ per year), rapid progression to CKD (CKDi25, defined as a decline in eGFR ≥25% of baseline while progressing from no CKD to CKD), and dialysis. By utilizing a bidirectional MR approach, we aimed to provide a more comprehensive understanding of the complex relationships between inflammatory factors and dynamic changes in renal function.

## 2 Method

### 2.1 Data source of inflammatory markers

We obtained genome-wide association analysis (GWAS) data for circulating concentrations of 41 inflammatory factors from meta-analyses involving 8293 individuals from three independent population cohorts: the Cardiovascular Risk in Young Finns Study (YFS), FINRISK1997, and FINRISK2002 (27). A total of 48 cytokines were measured in YFS and FINRISK2002, following the instructions of the Pro Human Cytokine assay kit (Bio-Rad, Hercules, California, USA). Seven cytokines with missing values exceeding 90% were removed, and 17 cytokines that overlapped with those in FINRISK2002 and YFS were searched for in FINRISK1997. Cytokines were quantified from EDTA plasma in FINRISK1997, from heparin plasma in FINRISK2002, and from serum in YFS. In the original study, a series of rigorous interventions were employed to standardize the expression of cytokine effect sizes, ensuring robust and reliable results. To begin with, cytokine distributions were meticulously normalized through an inverse transformation process. Subsequently, the transformed phenotypes underwent meticulous adjustments for significant genetic principal components, such as age, sex, and body mass index. To ensure the adherence to normal distribution assumptions, another round of meticulous inverse transformation was performed on the model residuals. Subsequently, genome-wide association testing was conducted with the Snptest2 software v.2.5beta. Meta-analyses were performed using METAL software (v.2011-03-25). We have placed these cytokine source details in the **Supplementary Table**.

### 2.2 Data source of CKD and kidney function

We utilized data from the CKDGen Consortium for instrumental variables associated with CKD and eGFR, which were the primary outcomes. CKD was defined as eGFR < 60 ml/min/1.73m^2^, and the GWAS data for CKD were obtained from a meta-analysis involving 23 cohorts of European origin comprising 41,395 patients and 439,303 controls (28). eGFR GWAS data were obtained from meta-analyses conducted in the UK Biobank (n = 436,581, European origin) and the CKDGen consortium (n = 765,348, predominantly European origin) (29). In the UK Biobank, serum creatinine was measured using a Beckman Coulter AU5800 analysis and substituted into the Chronic Kidney Disease Epidemiology Collaboration (CKD-EPI) formula to calculate eGFR (30, 31). For individuals aged less than 18 years, the Schwartz formula was used instead (32). Additionally, to assess the relationship between inflammatory factors and dynamic changes in renal function, we included three cohort studies as endpoints. Rapid3 (34,874 cases and 107,090 controls) and CKDi25 (19,901 cases and 175,244 controls) data were obtained from the CKDGen Consortium and a GWAS meta-analysis of 42 studies primarily conducted in UK Biobank with European ancestry (33). Dialysis data were obtained from the Finngen database (r8) of Finns, including 954 cases and 330,300 controls (34).

### 2.3 Filtering of single nucleotide polymorphisms (SNPs)

Selection of appropriate SNPs is critical for the success of MR analysis. The fundamental assumption of MR requires that all SNPs strongly and independently predict exposure at the genome-wide significance level. In our study, we used 41 inflammatory factor-associated SNPs as instrumental variables for exposure. However, a strict threshold of 5 × 10^-8^ would have excluded the majority of SNPs. Therefore, we set a relatively lenient but still strongly significant threshold of 5 × 10^-6^, based on previous studies (35), to include most inflammatory modulator-associated SNPs with an R^2^ < 0.001 and kb = 10,000 to eliminate chain imbalance. For CKD and renal function-related phenotypes, we used a threshold of 5 × 10^-8^ for all instrumental variables except for Rapid3, for which we used a threshold of 5 × 10^-6^ due to fewer eligible SNPs, and we similarly set R^2^ < 0.001 and kb = 10,000 to eliminate linkage disequilibrium. Additionally, we removed weakly validated SNPs with F values less than 10 (equation: F = β^2^_exposure_/SE^2^_exposure_) to ensure the strength of association between instrumental variables and exposure factors. These screening conditions ensure the credibility of the results of our study.

### 2.4 Two-sample MR

**Figure 1:**
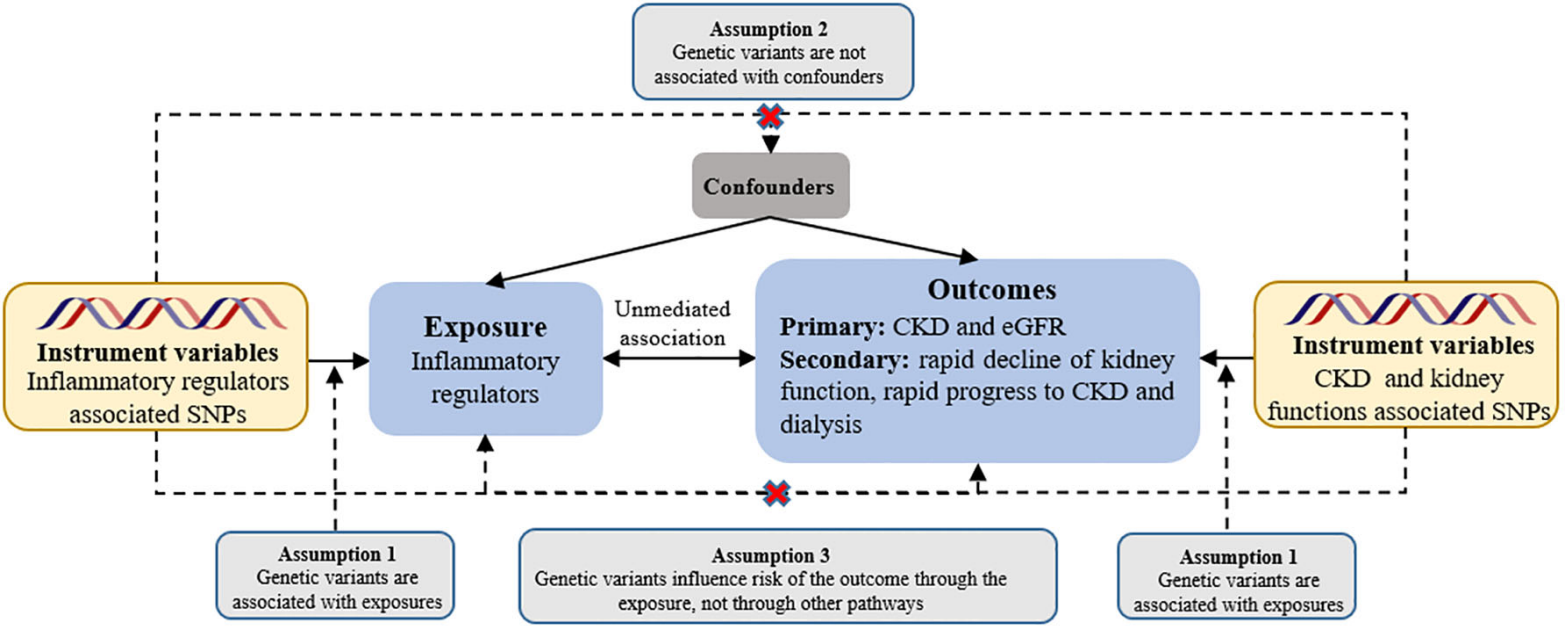
Assumptions of a mendelian randomization analysis for inflammatory regulators and risk of CKD. Broken lines represent potential pleiotropic or direct causal effects between variables that would violate Mendelian randomization assumptions. eGFR, estimated glomerular filtration rate.

We used two-sample MR analysis to assess the causal effect of systemic inflammatory modifiers on CKD and renal function. Instrumental estimates of MR for individual SNPs were derived using instrumental variable ratios. Assuming valid instruments without pleiotropy, we performed inverse variance-weighted fixed effects (IVW-FE) MR between instrumental estimates and standard errors (36), following the three main assumptions of MR (see **Figure 1**). To address horizontal pleiotropy, caused by genetic variation influencing outcomes through pathways other than the exposure, we used multiple methods: inverse variance-weighted random effects (IVW-RE), weighted median (WM), and MR-Egger approaches (37, 38). Moreover, we performed a comprehensive sensitivity analysis employing multiple methods to ensure the robustness of our findings. These methods included the heterogeneity test, horizontal pleiotropy assessment, funnel plot analysis, and leave-one-out analysis of IVW-RE. In the leave-one-out analysis, we systematically excluded one variant at a time to examine its impact on the results. To assess the individual instrumental variables, we employed the instrumental variable ratio (Wald) estimator. This estimator allowed us to evaluate the strength and validity of each instrument used in our instrumental variable analysis. To assess heterogeneity in individual causal effects, we calculated Q-statistics, with p-values < 0.05 indicating heterogeneity (39). In addition to the aforementioned approaches, we also employed a complementary WM method. This method ensures a reliable MR estimate by validating at least 50% of the inverse-variance and ranking its weighted variance. To tackle horizontal pleiotropy, we employed MR pleiotropy residual sum and outlier (MR-PRESSO) test for correction (40). When multiple sets of data are processed and compared simultaneously, there is a risk of encountering false positive results due to random effects. To mitigate this potential issue, we employed the Bonferroni correction test to assess the strength of the causal relationship between the exposure and outcome variables. A significance level of P < 0.05 was considered as suggestive evidence for a causal relationship. We used a significance threshold of 0.0045 for chemokines (11 factors), 0.0056 for growth factors (9 factors), 0.003125 for interleukins (16 factors), and 0.01 for other types (5 factors).

## 3 Results

### 3.1 Selection of instrumental variables

In our study, we initially screened 41 inflammatory factors for instrumental variables separately, resulting in a total of 452 SNPs that met our set screening criteria, all of which exhibited strong association strength (F-statistics range of 11-789). In the reverse MR analysis, we screened 5 instrumental variables related to CKD, resulting in 60390 SNPs meeting the significance range. After removing chain imbalance, we retained 514 SNPs. Further calculation of the F-statistics of these SNPs revealed that only 1 SNP, namely rs13329952, was strongly correlated with the exposure factor for Rapid3 with an F-statistic of 14, and rs12922822 was identified as an instrumental variable for CKDi25 with an F-statistic of 27. Additionally, we identified 23, 340, and 5 instrumental variables that exhibited strong association with CKD, eGFR, and dialysis, with F-statistics ranging from 25-693. The details of these SNPs are shown in the **Supplementary Table**.

### 3.2 Causal link between inflammatory factors and CKD

**Figure 2:**
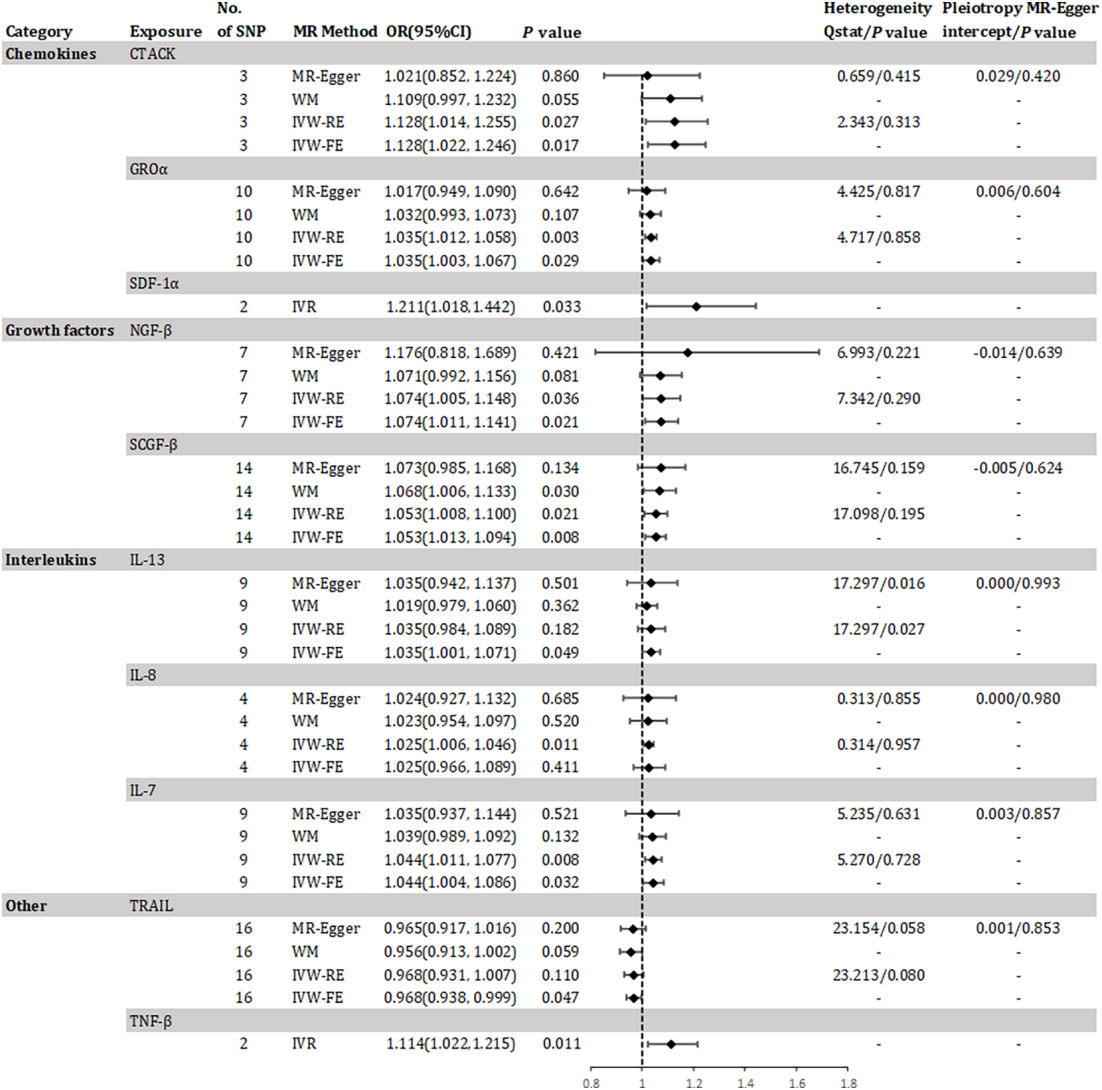
Odds ratio for association of genetically predicted systemic inflammatory regulators with chronic kidney disease. CTACK, cutaneous T-cell attracting; GROa, growth regulated oncogene alpha; SDF-1a, stromal-cell-derived factor 1 alpha; NGF-B, beta-nerve growth factor; SCGF-B, stem cell growth factor beta; IL, interleukin; TNF-β, tumor necrosis factor beta; TRAIL, TNF-related apoptosis inducing ligand; MR, mendelian randomization; CI, confidence internal; OR, odds ratio; IVW-FE, inverse-variance weighted fixed-effects MR; IVW-RE, inverse-variance weighted random-effects MR; WM, weighted median; IVR, instrumental variable ratio (Wald) estimator; SNP, single nucleotide polymorphism. *P* value for heterogeneity based on Cochran's Q statistic for IVW, and Rücker's Q for MR-Egger.

The study found evidence of a causal link between 10 inflammatory factors and an increased risk of developing CKD (as shown in **Figure 2**). The IVW-RE method for genetic prediction revealed that higher levels of cutaneous T-cell attracting (CRACK) (OR=1.128, 95% CI 1.014 - 1.255, *P* = 0.027), growth regulated oncogene alpha (GROα) (OR=1.035, 95% CI 1.012 - 1.058, *P* = 0.003), beta-nerve growth factor (NGF-β) (OR=1.074, 95% CI 1.005 - 1.148, *P* = 0.036), stem cell growth factor beta (SCGF-β) (OR=1.053, 95% CI 1.008 - 1.100, *P* = 0.021), interleukin-8 (IL-8) (OR=1.025, 95% CI 1.006 - 1.046, *P* = 0.011), and interleukin-7 (IL-7) (OR=1.044, 95% CI 1.011 - 1.077, *P* = 0.008) were associated with an increased risk of CKD, and the results were similar with the IVW-FE, MR-Egger and weighted median analyses. The results from IVW-FE showed that higher levels of IL-13 were associated with a higher risk of CKD (OR=1.035, 95% CI 1.001 - 1.071, *P* = 0.049). We identified 2 of 5 SNPs for TNF-β and 2 of 10 SNPs for stromal-cell-derived factor 1 alpha (SDF-1α) associated with CKD. The study found that higher levels of SDF-1α (OR=1.211, 95% CI 1.018 - 1.442, *P* = 0.033) and TNF-β (OR=1.114, 95% CI 1.022 - 1.215, *P* = 0.011) were associated with an increased risk of CKD using Wald analysis. However, the IVW-FE analysis revealed that lower levels of TNF-related apoptosis inducing ligand (TRAIL) were associated with a higher risk of CKD (OR=0.968, 95% CI 0.938 - 0.999, *P* = 0.047), which was observed in the IVW-RE, MR-Egger and weighted median analyses that were consistent. Although there was some evidence of heterogeneity based on Q-statistics in the IL-13 analysis, no evidence of heterogeneity was found in the analysis of other inflammatory factors. **Supplementary eFigure 1** displays a scatter plot depicting the association between genetically predicted inflammatory regulators and CKD. Horizontal pleiotropy was not detected through MR-Egger and MR-PRESSO tests (*P >*0.05). Additionally, the leave-one-out test did not identify any variants that significantly influenced the overall outcome (**Supplementary eFigure 2**). The funnel plot displayed a symmetrical distribution (**Supplementary eFigure 3**). Results of the Bonferroni correction test demonstrated that higher levels of GROα remained significantly associated with an increased risk of CKD.

### 3.3 Causal link between inflammatory factors and eGFR

**Figure 3:**
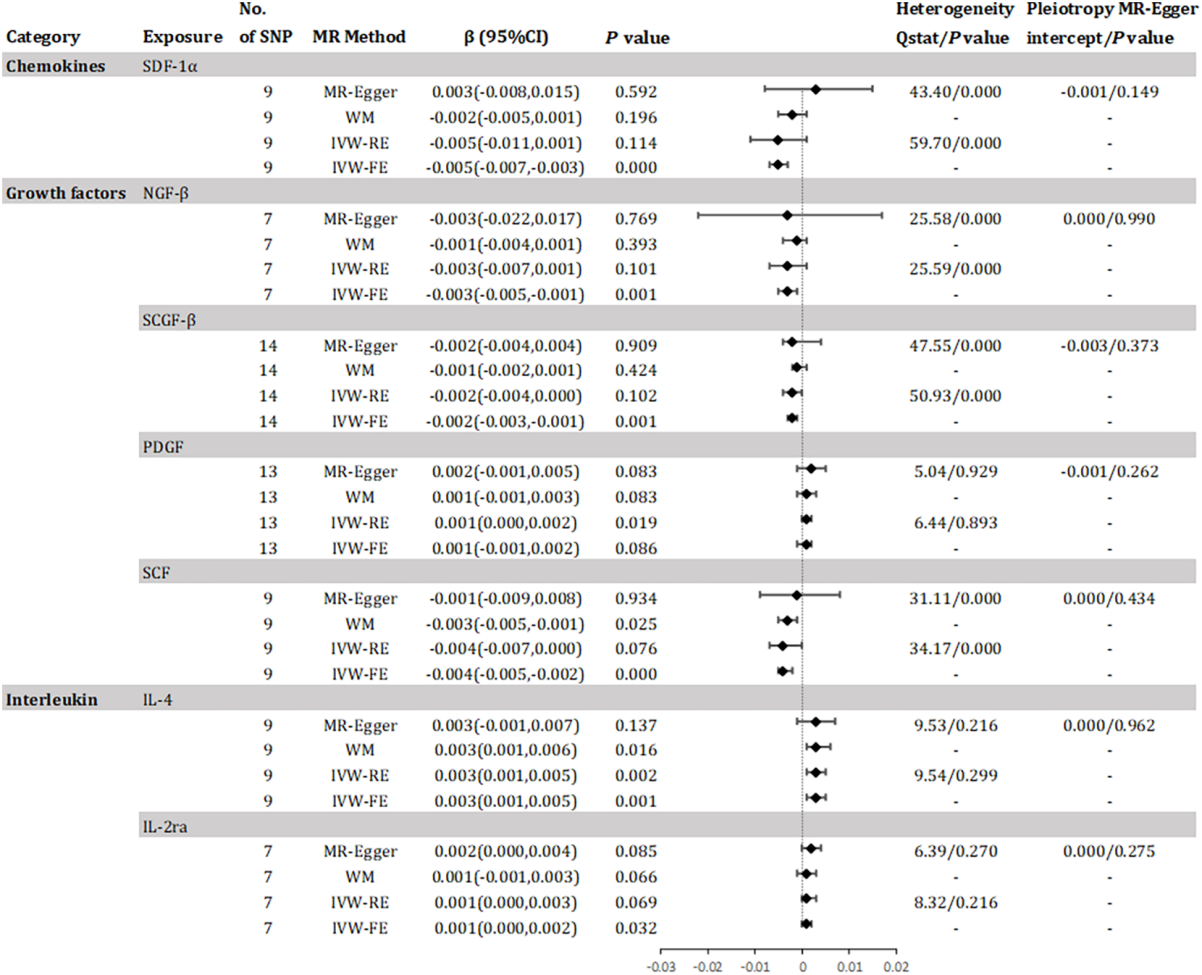
Effect for association of genetically predicted systemic inflammatory regulators with estimated glomerular filtration rate. SCGF-B, stem cell growth factor beta; NGF-β, beta-nerve growth factor; IL, interleukin; SDF-1a, stromal-cell-derived factor 1 alpha; SCF, stem cell factor; PDGF, platelet-derived growth factor BB; MR, mendelian randomization; CI, confidence internal; OR, odds ratio; IVW-FE, inverse-variance weighted fixed-effects MR; IVW-RE, inverse-variance weighted random-effects MR; WM, weighted median; SNP, single nucleotide polymorphism. *P* value for heterogeneity based on Cochran's Q statistic for IVW, and Rücker's Q for MR-Egger.

The study found evidence of a causal link between 10 inflammatory factors and an increased risk of declined eGFR (as shown in **Figure 3**). The IVW method for genetic prediction revealed that 1 SD-increase in SDF-1α(β = -0.005, 95% CI -0.007 - -0.003, *P* = 0.000), NGF-β (β = -0.003, 95% CI -0.005 - -0.001, *P* = 0.001), SCGF-β (β = -0.002, 95% CI -0.003 - -0.001, *P* = 0.001) and stem cell factor (SCF)(β = -0.004, 95% CI -0.005 - -0.002, *P* = 0.000) by the allele were associated with decreased eGFR. The difference is that a genetically predicted 1 SD-increase in platelet-derived growth factor BB (PDGF) (β = 0.001, 95% CI 0.000 – 0.002, *P* = 0.019), IL-4 (β = 0.003, 95% CI 0.001 – 0.005, *P* = 0.002) and IL-2ra (β = 0.001, 95% CI 0.000 - 0.002, *P* = 0.032) by the allele were associated with increased eGFR. The analysis of SDF-1α, NGF-β, SCGF-β and SCF showed some evidence of heterogeneity based on Q-statistics, while no evidence of heterogeneity was found in the analysis of other inflammatory factors. **Supplementary eFigure 4** provided scatter plots showing the relationship between these inflammatory regulators and eGFR. MR-Egger and MR-PRESSO tests did not show evidence of horizontal pleiotropy (*P >*0.05). The leave-one-out test did not identify any associated variants that strongly influenced the overall results (**Supplementary eFigure 5**), and the funnel plot showed overall symmetry (**Supplementary eFigure 6**). According to the Bonferroni correction test, higher levels of IL-4 maintained a strong causal relationship with elevated eGFR.

### 3.4 Causal link between inflammatory factors and dynamic changes in renal function

**Figure 4:**
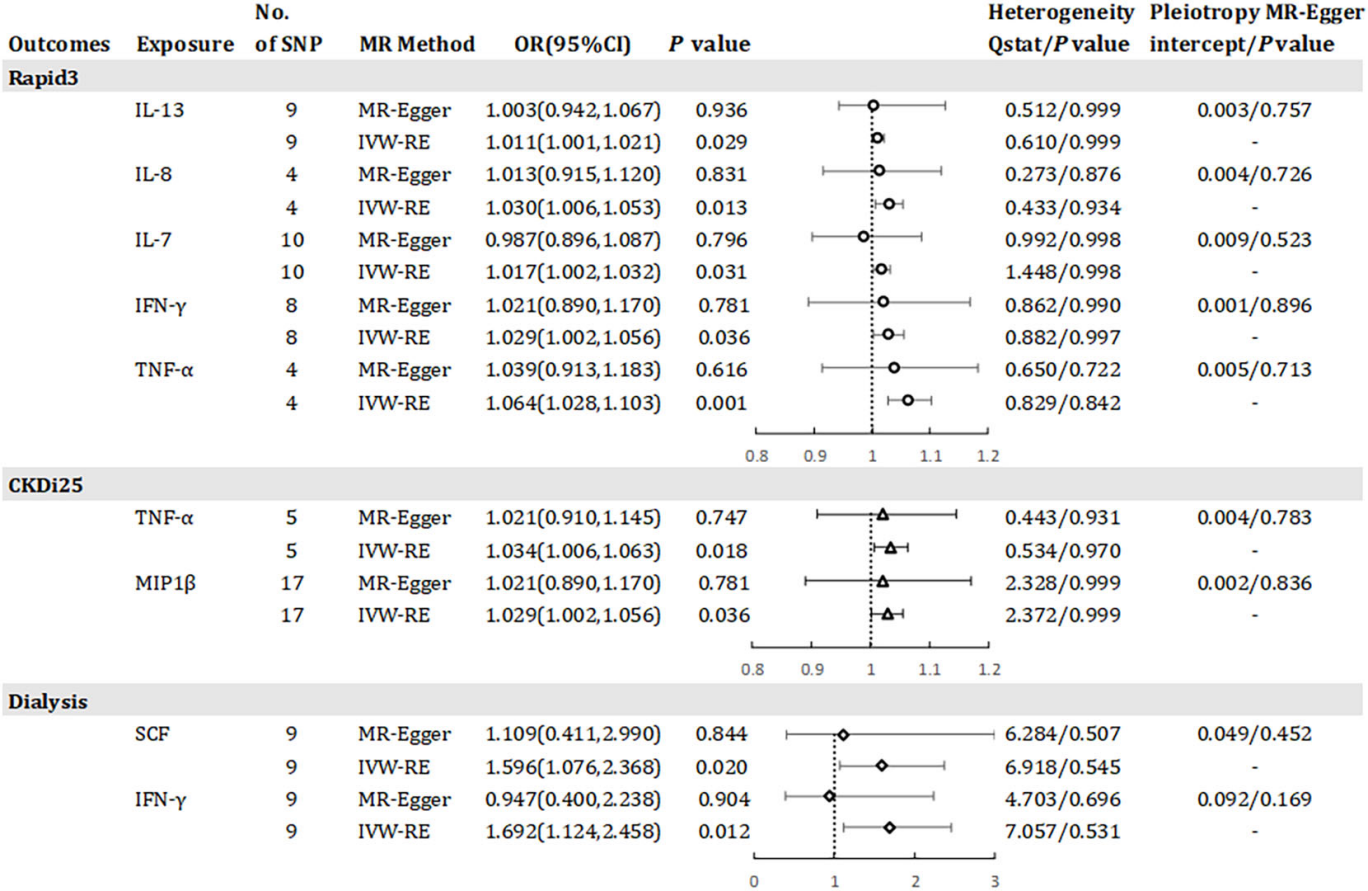
Odds ratio for association of genetically predicted systemic inflammatory regulators with Rapid3, CKDI25 and dialysis. IL, interleukin; TNF-a, tumor necrosis factor alpha; IFN-y, interferon gamma; MIP1bβ, macrophage inflammatory protein 1 beta; SCF, stem cell factor; MR, mendelian randomization; CI, confidence internal; OR, odds ratio; IVW-RE, inverse-variance weighted random- effects MR; SNP, single nucleotide polymorphism; Rapid3, rapid decline in renal function, i.e., a decline in eGFR of more than 3 mL/min/1.73 m^2^ per year; CKDI25, rapid progression to chronic kidney disease (CKD), i.e., a decrease in eGFR ≥ 25% of baseline, along with progression from no CKD to CKD. P value for heterogeneity based on Cochran's Q statistic for IVW, and Rücker's Q for MR- Egger.

Results has revealed a causal relationship between seven inflammatory factors and changes in renal function, with five factors associated with Rapid3 risk, two with CKDi25 risk, and two with dialysis risk (see **Figure 4**). A genetically predicted 1 SD-increase in IL-13 (OR = 1.011, 95% CI 1.001 - 1.021, *P* = 0.029), IL-8 (OR = 1.030, 95% CI 1.006 - 1.053, *P* = 0.013) and IL-7 (OR = 1.017, 95% CI 1.002 - 1.032, *P* = 0.031) by the allele was associated with a higher risk of Rapid3. Macrophage inflammatory protein 1 beta (MIP1β) was associated with an elevated risk of CKDi25 (OR = 1.029, 95% CI 1.002 - 1.056, *P* = 0.036) and SCF was associated with an elevated risk of dialysis (OR = 1.596, 95% CI 1.076 – 2.368, *P* = 0.020). TNF-α was associated with an elevated risk of CKDi25 (OR = 1.034, 95% CI 1.006 - 1.063, *P* = 0.018) and Rapid3 (OR = 1.064, 95% CI 1.028 – 1.103, *P* = 0.001). Interferon gamma (IFN-γ) was associated with an increased risk of dialysis (OR = 1.692, 95% CI 1.124 – 2.458, *P* = 0.012) and Rapid3 (OR = 1.029, 95% CI 1.002 – 1.056, *P* = 0.036). We found no evidence of heterogeneity in the course of our analysis. Scatter plots of the relationships between these inflammatory regulators and Rapid3, CKDi25, and dialysis are provided in **Supplementary eFigures 7, 10, 13**, respectively. MR-Egger and MR-PRESSO tests did not show evidence of horizontal pleiotropy (*P >*0.05). The leave-one-out test did not identify any associated variants that strongly influenced the overall results (**Supplementary eFigures 8, 11, 14**), and the funnel plot showed overall symmetry (**Supplementary eFigures 9, 12, 15**). The results of the Bonferroni correction test showed that higher levels of TNF-α maintained a strong causal relationship with higher risk of Rapid3.

### 3.5 Reverse analysis

**Figure 5:**
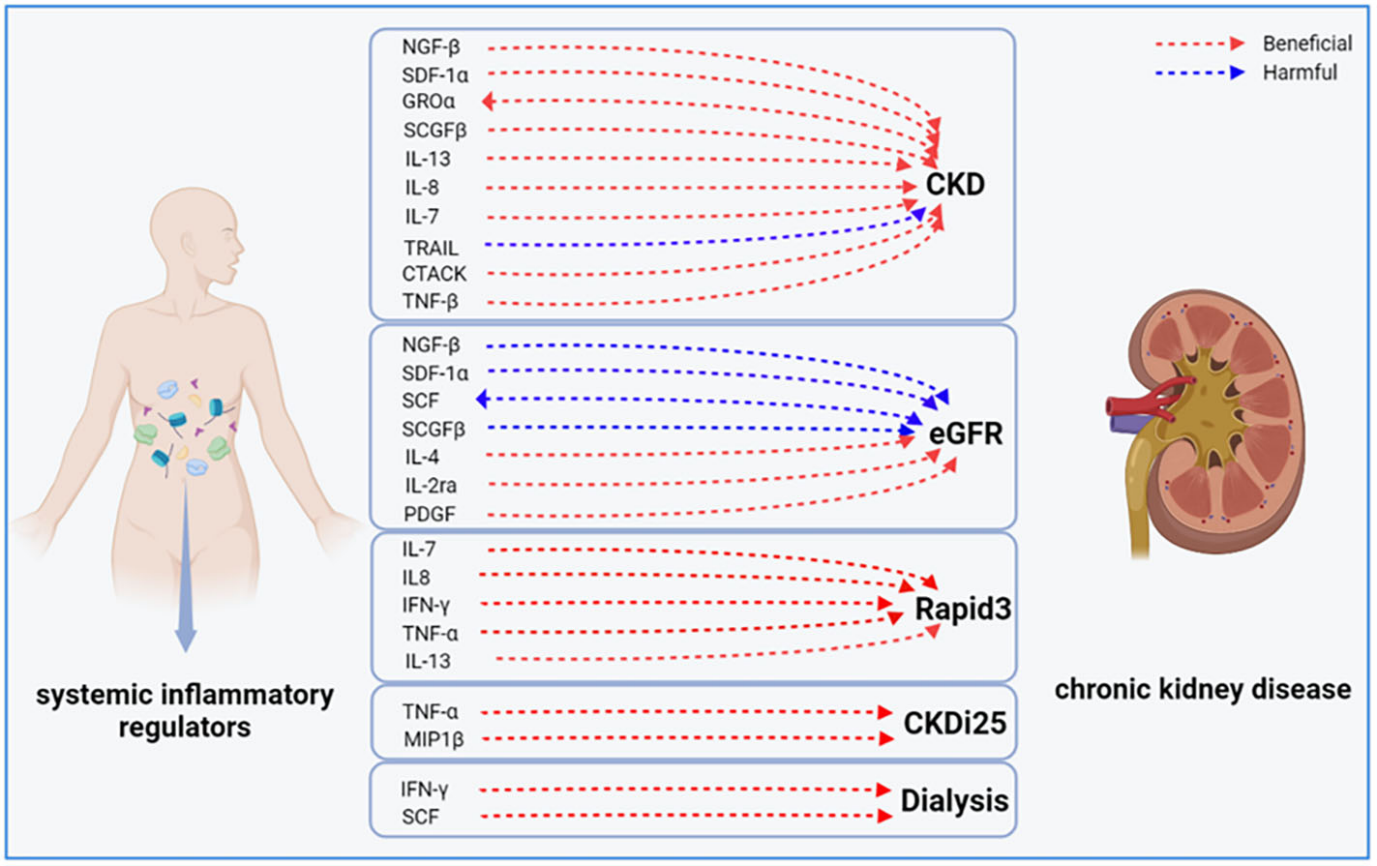
Bidirectional causal link between systemic inflammatory factors and chronic kidney disease. CKD, chronic kidney disease; eGFR, estimated glomerular filtration rate; CKDI25, rapid progression to CKD, i.e., a decrease in eGFR ≥ 25% of baseline, along with progression from no CKD to CKD; Rapid3, rapid decline in renal function, i.e., a decline in eGFR of more than 3 mL/min/1.73 m^2^ per year.

In this study, we identified causal associations between 17 inflammatory factors and various CKD outcomes through forward analyses. We also conducted a reverse study to determine the genetic association between CKD and these 17 inflammatory factors. The ivw-RE analysis revealed that CKD may lead to higher levels of GROα (β = 0.144, 95% CI 0.010 - 0.278, *P* = 0.036), and reduced eGFR may lead to elevated levels of SCF (β = -1.998, 95% CI -2.773 - -1.223, *P* = 0.000). However, we did not observe clear associations of other inflammatory factors with Rapid3, CKDi25, and dialysis risk (**Supplementary eFigures 16–18**). **Figure 5** illustrates a bidirectional causal relationship between inflammatory factors and CKD. During the analysis, we found no evidence of horizontal pleiotropy (*P >*0.05).

## 4 Discussion

To our knowledge, our study represents the first large-scale and comprehensive MR investigation to explore the genetic causal relationship between systemic inflammatory regulators and CKD. Previous studies have mainly focused on the cellular or animal level, examining local inflammation in renal tissues or cells rather than the systemic inflammatory response of the organism (41, 42). Observational studies based on clinical settings are often limited by confounding factors and reverse causation bias, leading to distorted causal relationships between variables. By integrating GWAS data from multiple large populations, our study identified 17 inflammatory factors that are genetically associated with various renal outcomes. Specifically, we found strong causal associations between GROα and CKD, IL4 and eGFR, and TNF-α and Rapid3. Subsequent analysis revealed that genetic susceptibility to CKD resulted in an increase in GROa and SCF. These findings highlight the genetic regulation of systemic inflammatory factors by chronic kidney injury.

In this study, we established links between inflammatory factors and multiple outcomes related to CKD, with some inflammatory factors playing important regulatory roles in various outcomes, consistent with previous research. For instance, elevated NGF-β, SDF-1α, and SCGFβ were not only associated with a higher risk of CKD, but also had the potential to decrease eGFR; increased IL7, IL8, and IL13 not only contributed to the likelihood of CKD, but also may have contributed to Rapid3; TNF was associated with both CKD and CKDi25; increased IFN-γ not only contributed to a higher risk of Rapid3, but also may be causally associated with dialysis. There is ample evidence to suggest that unresolved inflammatory processes can lead to renal fibrosis and eventual ESKD, as seen in conditions such as Alport syndrome, autosomal dominant polycystic kidney disease (ADPKD), IgA nephropathy, and focal segmental glomerulosclerosis (43). Our results further validate the direct causal association between systemic inflammation and renal function.

IL are the most diverse and extensively studied family of cytokines with a complex impact on renal function (44). Within this family, IL-8 is a multifunctional factor that specifically attracts neutrophils to inflamed tissues, triggering degranulation, superoxide anion production, respiratory burst, and promoting the release of inflammatory mediators. IL-8 has been established as a critical factor in inducing CKD (45). Studies have demonstrated that not only is IL-8 elevated in adult CKD patients (46), but also in pre-dialysis CKD children compared to healthy controls (47, 48). Meanwhile, IL8 has emerged as an independent risk predictor of CKD and its associated vascular damage. For instance, in patients with type 2 diabetes mellitus, abnormally elevated IL8 levels were found to be associated with a 1.41-fold increased risk of urinary protein (49). Moreover, in patients on hemodialysis, proteomics-detected IL8 was found to predict all-cause mortality risk ratios of up to 1.17, with an elevated cardiovascular all-cause mortality risk ratio of up to 1.34 (50). These predicted risk values align closely with the findings from our study. Previous investigations have demonstrated that chemokines with a sequence similar to IL-8 were highly expressed in rodent models with renal cysts (51). In two different ADPKD cell lines (WT9-7 and WT9-12), IL-8 secretion and expression were found to be significantly increased compared to normal human renal cortical epithelial cell lines. Cell proliferation, mediated by IL-8 signaling, was inhibited by antagonists or siRNAs targeting the IL-8 receptor, and *in vitro* cystogenesis was attenuated after blocking IL-8 receptor signaling, thus confirming the promoting effect of IL-8 on CKD development (52). However, a recent study further elucidated the effects of different types of IL-8 release on renal structure. In glomerulonephritis specimens, the expression of 72-AA type IL-8 was found to increase in podocytes, whereas 77-A type IL-8 was predominantly expressed in podocytes and interstitial vascular endothelial cells from healthy kidneys and may be associated with preserving glomerular structure (53). Thymocyte differentiation antigen 1 (Thy-1) regulates several fundamental fibroblast functions associated with fibrogenesis and is detectable in serum and urine as soluble Thy-1 (sThy-1). Serum creatinine is a significant and independent predictor of sThy-1 levels. Pro-fibrotic cytokines, including IL-7, IL-13, and IL-8, promote the expression of Thy-1 genes and sThy-1 release from renal interstitial fibroblasts, leading to renal fibrosis and ultimately the development of CKD (54). Our findings highlight the detrimental effects of elevated levels of IL-13, IL-8, and IL-7 on renal function, further supporting the causal link between pro-fibrotic mediators and CKD.

While NGF is traditionally known for its involvement in neuronal injury, recent studies have identified its potential association with kidney function. For instance, single-cell RNA sequencing in a mouse model of renal fibrosis demonstrated an unexpected upregulation of NGF (55). SDF-1α, a CXC-type chemokine, is a known biomarker for angiogenesis, and the SDF-1/CXCR4 pathway plays a crucial role in the development of renal vasculature. Prior research has demonstrated increased renal expression of SDF-1 in rats with subtotal nephrectomy treated with angiotensin-converting enzyme inhibitors (56). Additionally, in a CKD rat model, therapy with peripheral blood-derived endothelial progenitor cells elevated SDF-1α expression at the protein level, suggesting a role for local SDF-1/CXCR4 signaling in preserving microvascular integrity and preventing renal fibrosis (57). However, systemic SDF-1 expression in CKD patients differs from *in vivo* models, with one study showing higher serum and SDF-1 levels in dialysis patients with chronic renal failure than in healthy controls (58), which aligns with our findings. Activation of CD56^bright^ natural killer cells produced by IFN-γ can play a critical role in the fibrotic process and progression to CKD (59). Clinical evidence suggests that serum IFN-γ levels are often elevated in individuals with CKD and uremia compared to healthy individuals (60, 61). Control of inflammatory factors may decrease body IFN-γ levels and thus regulate renal depletion and CKD-related plaque vulnerability (62, 63). Our findings are consistent with this, suggesting that controlling the body’s inflammatory response is not only protective against chronic renal impairment but also has additional benefits for other pathological responses such as vascular damage and nerve damage associated with CKD (64).

Our study has provided a noteworthy contribution to the understanding of the bidirectional association between systemic inflammation and CKD. The systemic inflammatory response is not only the initiating factor of CKD but is also a consequence of renal impairment. Previous research has primarily focused on the causes of CKD formation, neglecting the contribution of renal disease to the systemic inflammatory state (65–67). Renal disease is a potent modulator of normal gut microbiota composition and metabolism, which can lead to the production of toxins and inflammatory factors. In CKD, factors like gut dysbiosis, slow intestinal transit, low fiber intake, metabolic acidosis, intestinal ischemic edema, iron therapy, and frequent antibiotics exposure can promote the leakage of gut-derived factors (e.g., bacterial components, endotoxins, and intestinal metabolites) into circulation. This triggers immune activation and pro-inflammatory signaling (68, 69). CKD-associated impaired intestinal integrity can promote the leakage of intestinal metabolites, such as trimethylamine N-oxide, p-cresol, and indolol sulfate, as well as lipid peroxidation products, which can directly disrupt cholesterol metabolism, increase scavenger receptor expression, and promote foam cell formation. Moreover, renal disease leads to increased production of these potentially harmful compounds, which can pose a threat to systemic homeostasis, resulting in a range of cardiovascular complications closely linked to the inflammatory response (70). Oxidative stress and metabolic acidosis, which develop as glomerular filtration rate decreases, contribute to the proinflammatory state associated with CKD (71). The impaired nuclear factor erythroid 2-related factor 2 (Nrf2) system plays a pivotal role in this intricate process. Its activity is influenced by factors such as the etiology of renal disease, comorbidities, CKD stage, and the severity of uremic toxin accumulation and inflammation. Notably, early stages of CKD and rapid disease progression are associated with heightened Nrf2 system activation, while in later stages, a stronger inhibition of the Nrf2 system is observed (72). Therefore, systemic inflammation in CKD is not only a well-established risk factor for mortality, but also a potent catalyst for other complications. In the context of renal function decline, coupled with systemic inflammation, there exists a notable escalation in the susceptibility to disturbances in homeostasis. This heightened risk stems from the dual impact of reducing both the functional and structural tissue reserves within the body, while concurrently hampering the intricate interplay between organs that aids in recovery from both internal and external stressors (73).

Currently, there are ongoing drug investigations targeting inflammatory factors in CKD. An earlier proof-of-concept trial in lupus nephritis patients failed to establish the effectiveness of IL-6 inhibition alongside conventional therapy (74). Nevertheless, Pergola and colleagues conducted a RCT assessing the impact of ziltivekimab, a novel anti-IL-6 ligand antibody, in hemodialysis patients harboring polymorphisms predisposing them to IL-6-induced inflammation. Their findings revealed that treated patients exhibited not only improvements in inflammatory markers but also elevated serum albumin levels (75). Additionally, a case report demonstrated the protective effects of tocilizumab, utilized in patients with rheumatoid arthritis accompanied by AA amyloidosis and CKD, manifesting as diminishing proteinuria (76). Ongoing investigations are delving into the role of IL-1 inhibition in renal function. Buckley et al. observed a reduction in serum inflammatory markers following administration of recombinant human IL-1 receptor antagonists in individuals with cardiorenal syndrome (77). Conversely, Nowak et al. did not find enhancements in CKD-associated mineral and bone disorders or physical function after 12 weeks of rilonacept treatment, an IL-1 inhibitor (78). However, their subsequent study addressing vascular function demonstrated that the use of lixonacept in CKD was linked to improved brachial artery flow-mediated dilation and diminished systemic inflammation (79). Moreover, in the CANTOS trial, a human monoclonal antibody targeting IL-1β was employed in CKD patients, resulting in a reduction of major cardiovascular events (80). IL-8 exhibits a heightened specificity within renal pathophysiology, particularly in the context of diabetic nephropathy, and its expression inhibition could hold promise as a potential therapeutic target (81). IL-17A, a constituent of the IL-17 family, contributes to countering bacterial and fungal infections of the skin. It is also implicated in autoimmune and inflammatory disorders, along with its involvement in the pathogenesis of CKD (82, 83). A murine study demonstrated that blocking TNF-α in diabetic nephropathy led to reductions in albuminuria, serum creatinine, histopathological changes, and macrophage infiltration into the kidneys (84). Interferon, often employed as a non-renal therapeutic modality, has recently shown an emerging link to renal pathology. Notably, a case report highlighted a patient with hypereosinophilic syndrome who developed progressive renal failure and nephrotic-range proteinuria after a year of recombinant IFN-α-2b therapy. Renal injury reversed upon cessation of cytokine treatment (85). A similar nephrotic syndrome induced by IFN-β treatment was observed in a multiple sclerosis patient (86). These insights find partial clarification in Migliorini et al.’s investigation, which delineated distinct yet synergistic effects of INF-α and IFN-β on podocytes and parietal epithelial cells, ultimately culminating in glomerulosclerosis (87).

It is worth noting that the Bonferroni correction test may yield false negatives (88). Our study revealed that several systemic inflammatory factors were correlated with various phenotypes, but many of these correlations did not survive the Bonferroni correction test. This could be attributed to the intricate interplay between inflammatory mediators and the kidney, which is usually orchestrated by multiple factors. The pathogenesis of a single inflammatory factor may not be as significant as previously believed, and clusters of inflammatory factors may collaborate with each other to induce disease. By comprehending the pathophysiology of the combined response of these clusters of inflammatory factors in the body and their interaction with renal disease, we may gain a better understanding of the intricate mechanisms involved and develop targeted multi-inflammatory modulators in the future.

This pioneering MR study, utilizing recent pooled data, is the first to examine the causal connection between CKD and systemic inflammatory regulators. Unlike traditional observational studies, which are vulnerable to reverse causality bias due to the involvement of non-renal metabolic pathways in the systemic inflammatory response, this MR analysis minimizes confounding factors and reverse causality, providing a robust estimate of causality. Secondly, our study not only includes a GWAS cohort of CKD events alone but also incorporates multiple dynamic renal function indicators and phenotypes to synthesize the association between renal disease and inflammatory factors, which is more clinically relevant and informative. Finally, serum is one of the most readily accessible biological specimens in clinical practice, and the evidence from this study has broad implications for conducting future relevant clinical research.

It is equally important to acknowledge certain limitations in our study. First and foremost, the genetic data used for this analysis primarily came from individuals of European descent. As a result, the findings may not be applicable or generalizable to other ethnic groups, and caution should be exercised when extrapolating the results to diverse populations. Secondly, despite our best efforts to exclude SNPs that could be associated with potential confounders and conducting various sensitivity analyses under different assumptions, it is still possible that there are complex and multidirectional effects that might not be fully detected. Additionally, the use of instrumental variables from the GWAS meta-analysis prevented us from exploring potential stratification effects and nonlinear relationships, leaving room for further investigation into these aspects. We employed strict Bonferroni correction thresholds and other criteria, such as checking for evidence of pleiotropy, to identify the most reliable MR results. However, this strategy might lead to some false-negative results, although it minimizes the likelihood of identifying false-positive associations. Lastly, while MR analysis is a robust method for estimating causality, it should not be considered a substitute for RCTs. Therefore, the causality inferred from this study may not necessarily align with the results observed in RCTs. It is essential to conduct individual-based genetic observations and potentially incorporate RCTs in future research to further validate the causal relationships identified here.

In summary, our study using publicly available GWAS summary data and MR analysis has identified causal associations between 17 systemic inflammatory modulators and CKD outcomes, including bidirectional associations between GROα and SCF levels and renal function impairment. These findings offer promising genetic evidence for the development of targeted treatments for CKD across different stages.

## Data availability statement

The original contributions presented in the study are included in the article/**Supplementary Material**. Further inquiries can be directed to the corresponding author.

## Ethics statement

All the data utilized in this study have been sourced from the public domain, ensuring their accessibility and transparency. It is imperative to note that all participants involved in this research provided informed consent, thus upholding ethical standards. Furthermore, the study protocols underwent rigorous scrutiny and received the necessary approvals from the local ethical committees associated with each participant. These meticulous steps taken to adhere to ethical guidelines underscore the integrity and reliability of the findings presented in this study.

## Author contributions

HL: Conceptualization, Data curation, Formal analysis, Investigation, Methodology, and Writing - original draft. ML: Conceptualization, Formal analysis, Investigation, and Writing - original draft. CL: Data curation, Formal analysis; PH: Visualization, and Validation. SD: Funding acquisition and Supervision. AD: Investigation. MZ: Funding acquisition, Conceptualization, and Writing - review & editing. All authors contributed to this article and approved the submitted version.
