# Supplementary material for: Causal effects of systemic inflammatory regulators on chronic kidney diseases and renal function: a bidirectional Mendelian randomization study
Source: Front Immunol. 2023 Aug 30;14:1229636. doi: 10.3389/fimmu.2023.1229636 (PMC10498994; doi:10.3389/fimmu.2023.1229636)
Supplement: Supplementary file 1 [file DataSheet_1.docx]

**Supplementary Online Content**

**eFigure 1**. Scatter plot of systemic inflammatory regulators-associated SNPs with risk of chronic kidney disease.

**eFigure 2**. MR leave-one-out sensitivity analysis of systemic inflammatory regulators-associated SNPs with risk of chronic kidney disease.

**eFigure 3**. Funnel of systemic inflammatory regulators-associated SNPs with risk of chronic kidney disease.

**eFigure 4**. Scatter plot of systemic inflammatory regulators-associated SNPs with estimated glomerular filtration rate.

**eFigure 5**. MR leave-one-out sensitivity analysis of systemic inflammatory regulators-associated SNPs with estimated glomerular filtration rate.

**eFigure 6**. Funnel of systemic inflammatory regulators-associated SNPs with estimated glomerular filtration rate.

**eFigure 7**. Scatter plot of systemic inflammatory regulators-associated SNPs with risk of Rapid3.

**eFigure 8**. MR leave-one-out sensitivity analysis of systemic inflammatory regulators-associated SNPs with risk of Rapid3.

**eFigure 9**. Funnel of systemic inflammatory regulators-associated SNPs with risk of Rapid3.

**eFigure 10**. Scatter plot of systemic inflammatory regulators-associated SNPs with risk of CKDi25.

**eFigure 11**. MR leave-one-out sensitivity analysis of systemic inflammatory regulators-associated SNPs with risk of CKDi25.

**eFigure 12**. Funnel of systemic inflammatory regulators-associated SNPs with risk of CKDi25.

**eFigure 13**. Scatter plot of systemic inflammatory regulators-associated SNPs with risk of dialysis.

**eFigure 14**. MR leave-one-out sensitivity analysis of systemic inflammatory regulators-associated SNPs with risk of dialysis.

**eFigure 15.** Funnel of systemic inflammatory regulators-associated SNPs with risk of dialysis.

**eFigure 16.** Odds ratio for association of genetically predicted chronic kidney disease with systemic inflammatory regulators.

**eFigure 17.** Effect for association of genetically predicted estimated glomerular filtration rate with systemic inflammatory regulators.

**eFigure 18.** Odds ratio for association of genetically predicted Rapid3, CKDi25 and dialysis with systemic inflammatory regulators.

**
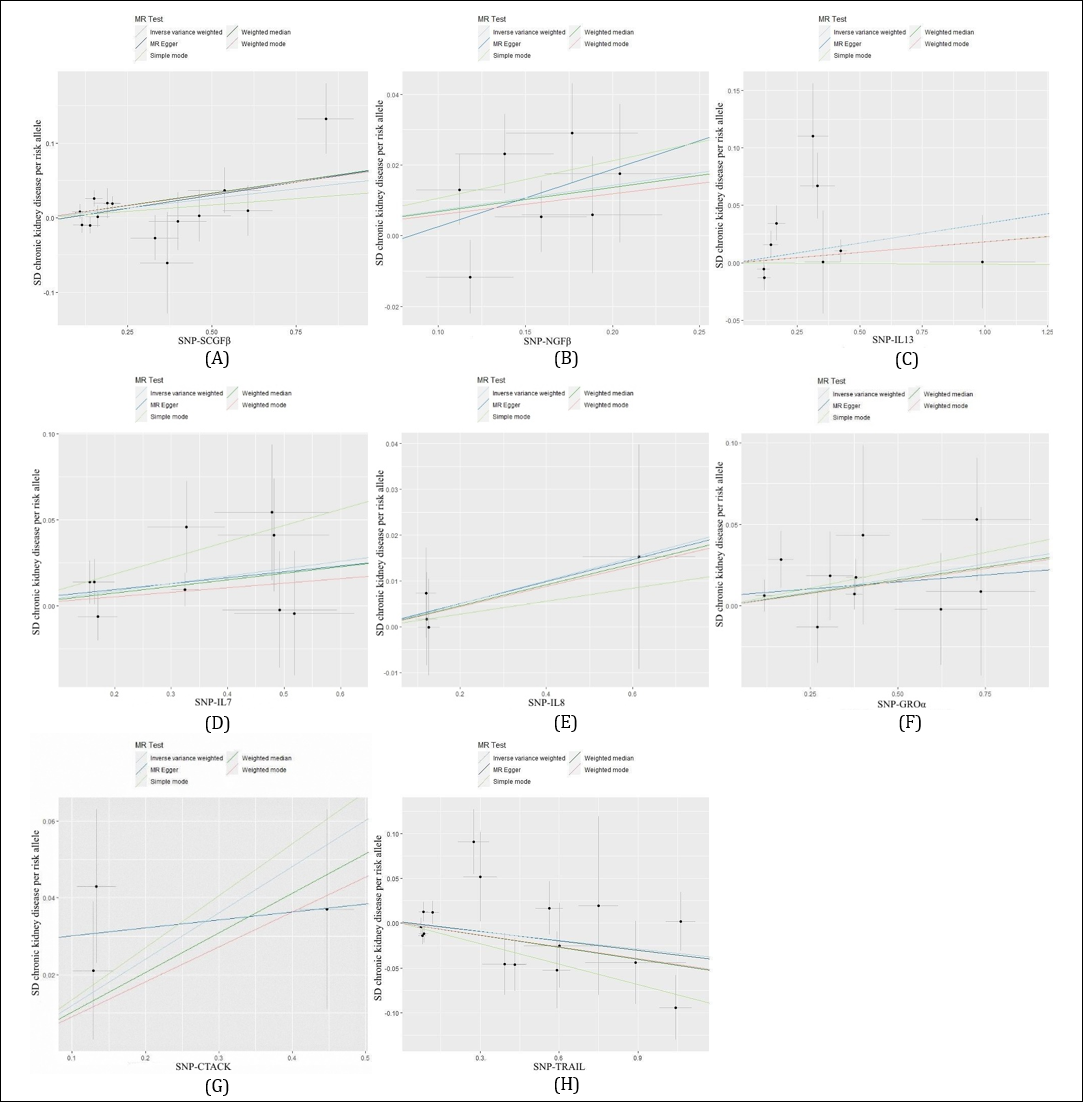
**

**eFigure 1**. Scatter plot of systemic inflammatory regulators-associated SNPs with risk of chronic kidney disease. The genetic relationship between SCGFβ, NGFβ, IL13, IL7, IL8, GROα, CTACK, TRAIL and chronic kidney disease, denoted as A to H, respectively. Abbreviations: CTACK, cutaneous T-cell attracting; TRAIL, TNF-related apoptosis inducing ligand; SCGFβ, stem cell growth factor beta; NGFβ, beta-nerve growth factor; GROa, growth-regulated oncogene-alpha; IL, interleukin; SD, standard deviation.

**
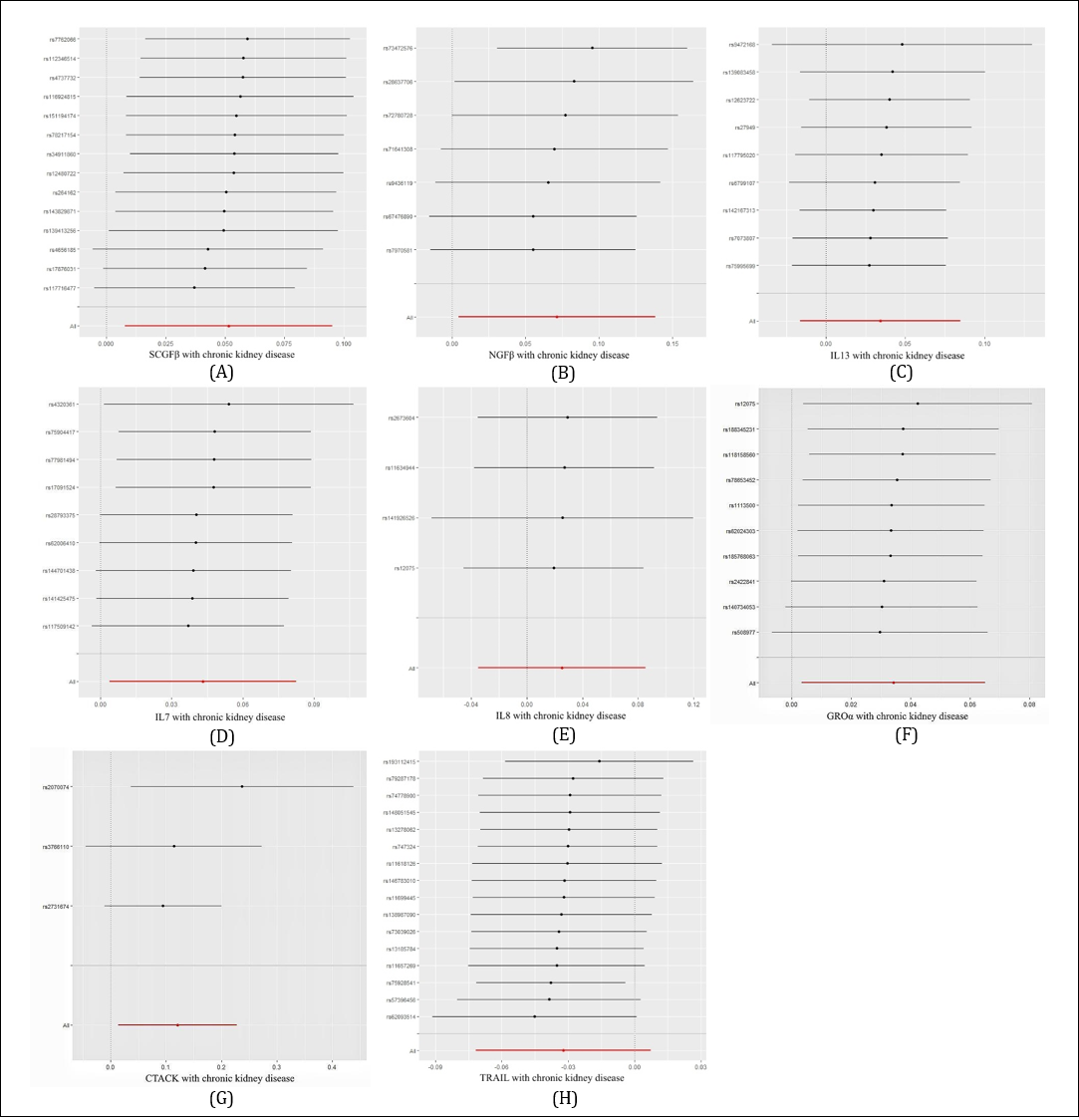
**

**eFigure 2**. MR leave-one-out sensitivity analysis of systemic inflammatory regulators-associated SNPs with risk of chronic kidney disease. The genetic relationship between SCGFβ, NGFβ, IL13, IL7, IL8, GROα, CTACK, TRAIL and chronic kidney disease, denoted as A to H, respectively. Abbreviations: CTACK, cutaneous T-cell attracting; TRAIL, TNF-related apoptosis inducing ligand; SCGFβ, stem cell growth factor beta; NGFβ, beta-nerve growth factor; GROa, growth-regulated oncogene-alpha; IL, interleukin.


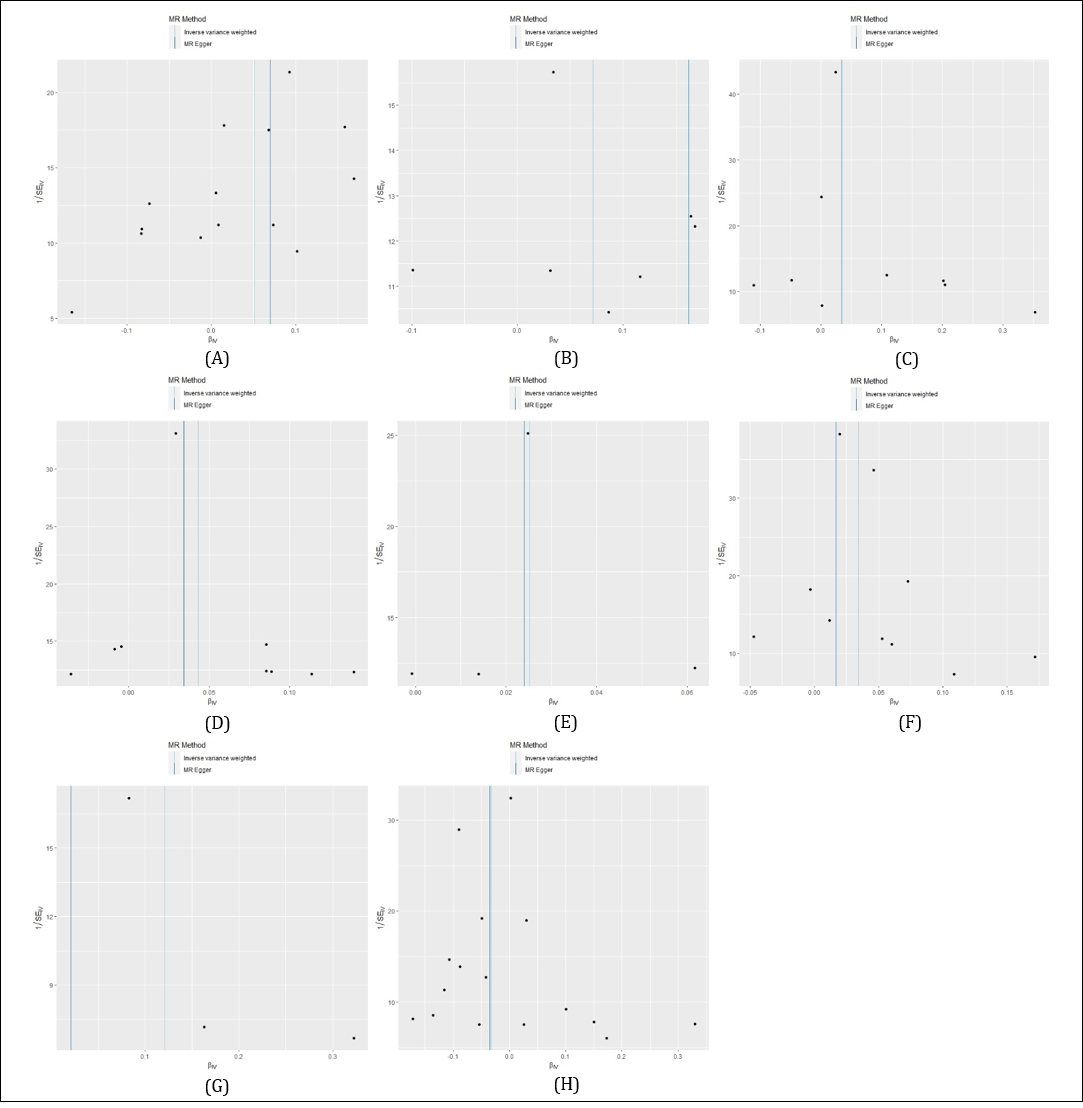


**eFigure 3**. Funnel of systemic inflammatory regulators-associated SNPs with risk of chronic kidney disease. The genetic relationship between SCGFβ, NGFβ, IL13, IL7, IL8, GROα, CTACK, TRAIL and chronic kidney disease, denoted as A to H, respectively. Abbreviations: CTACK, cutaneous T-cell attracting; TRAIL, TNF-related apoptosis inducing ligand; SCGFβ, stem cell growth factor beta; NGFβ, beta-nerve growth factor; GROa, growth-regulated oncogene-alpha; IL, interleukin.

**
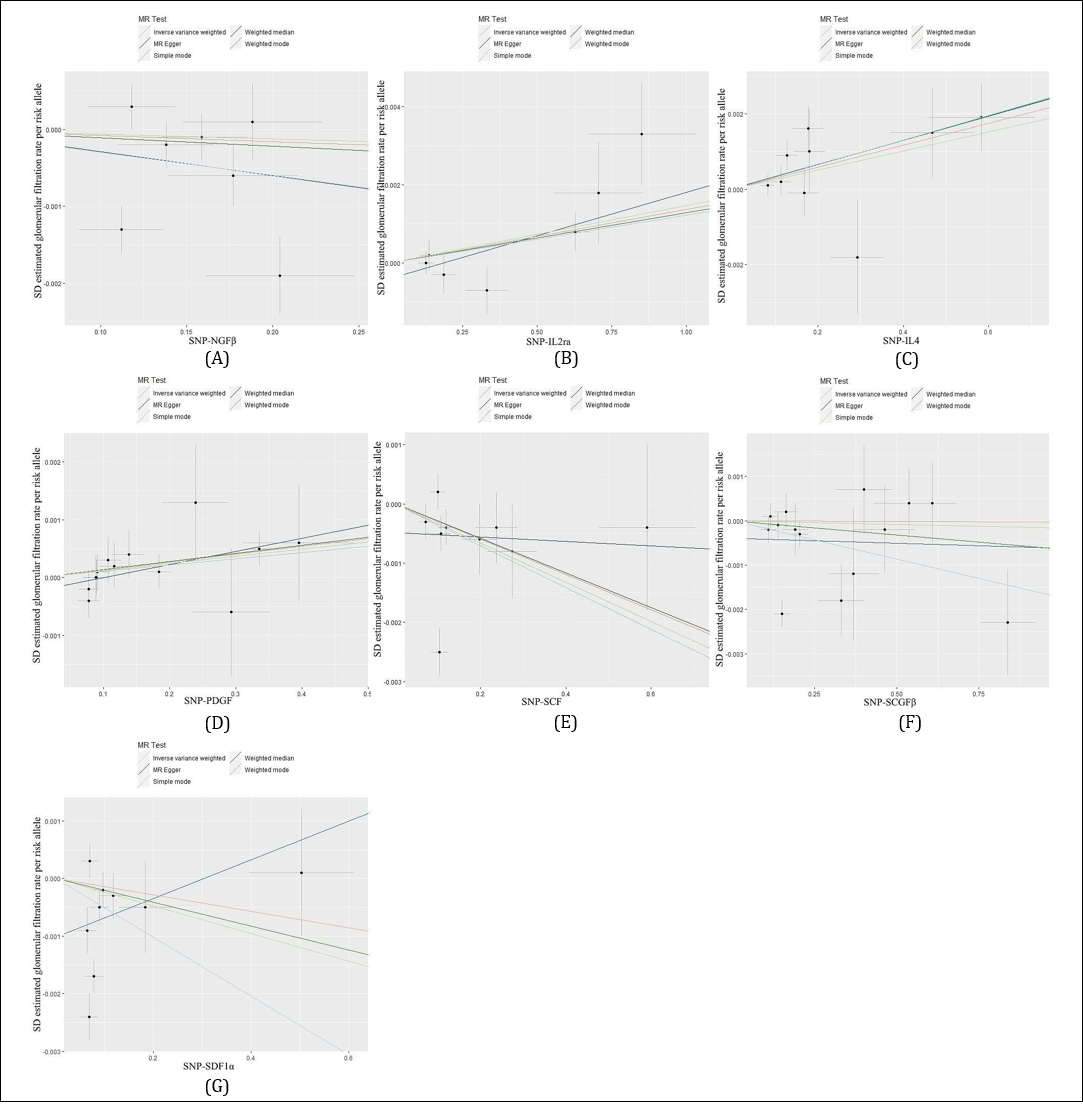
**

**eFigure 4**. Scatter plot of systemic inflammatory regulators-associated SNPs with estimated glomerular filtration rate.

The genetic relationship between NGFβ, IL2ra, IL4, PDGF, SCF, SCGFβ, SDF1α and estimated glomerular filtration rate, denoted as A to G, respectively. Abbreviations: SCGFβ, stem cell growth factor beta; NGFβ, beta-nerve growth factor; IL, interleukin; SDF1α, stromal-cell-derived factor 1 alpha; SCF, stem cell factor; PDGF, platelet-derived growth factor BB; SD, standard deviation.


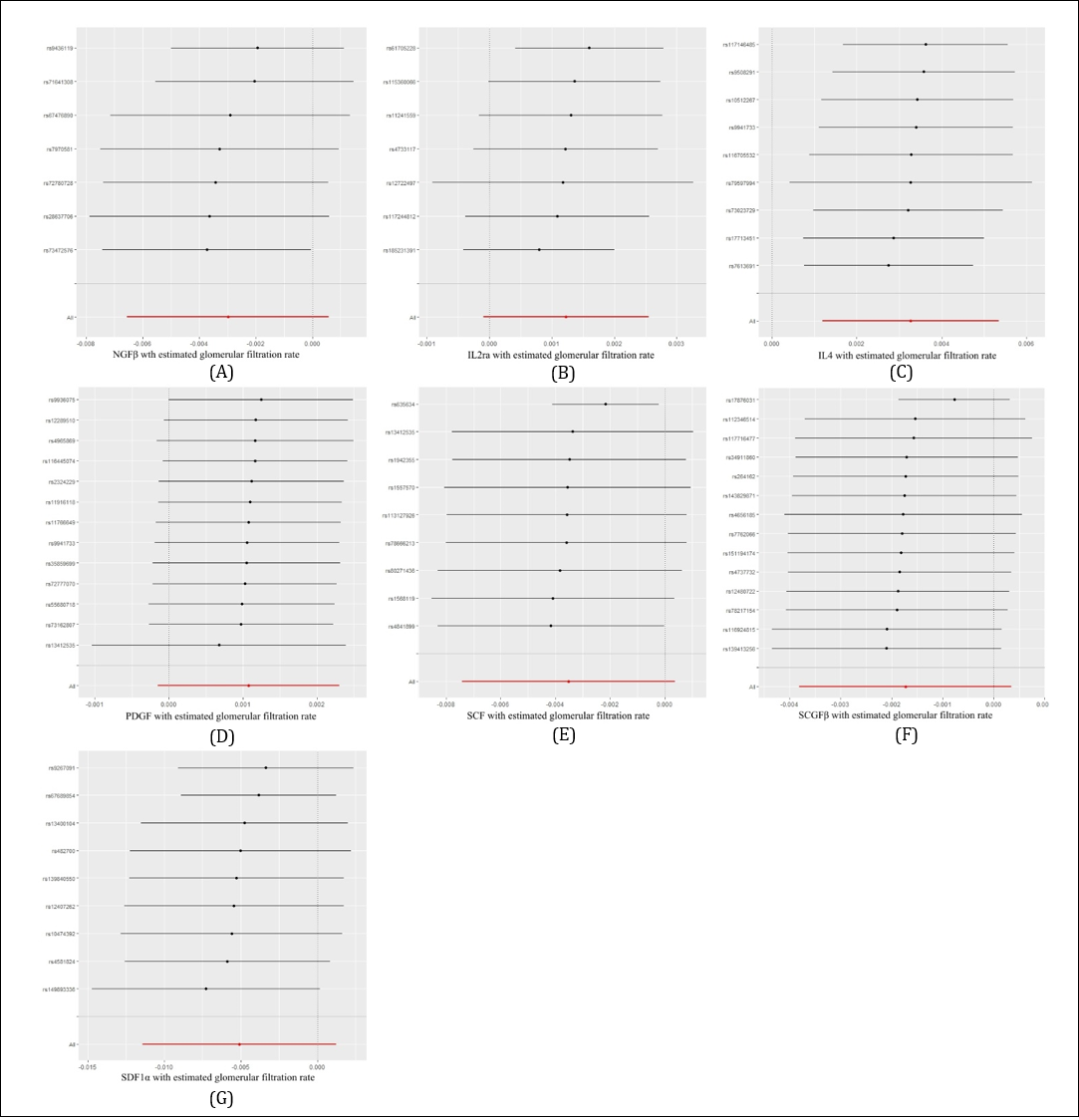


**eFigure 5**. MR leave-one-out sensitivity analysis of systemic inflammatory regulators-associated SNPs with estimated glomerular filtration rate. The genetic relationship between NGFβ, IL2ra, IL4, PDGF, SCF, SCGFβ, SDF1α and estimated glomerular filtration rate, denoted as A to G, respectively. Abbreviations: SCGFβ, stem cell growth factor beta; NGFβ, beta-nerve growth factor; IL, interleukin; SDF1α, stromal-cell-derived factor 1 alpha; SCF, stem cell factor; PDGF, platelet-derived growth factor BB.


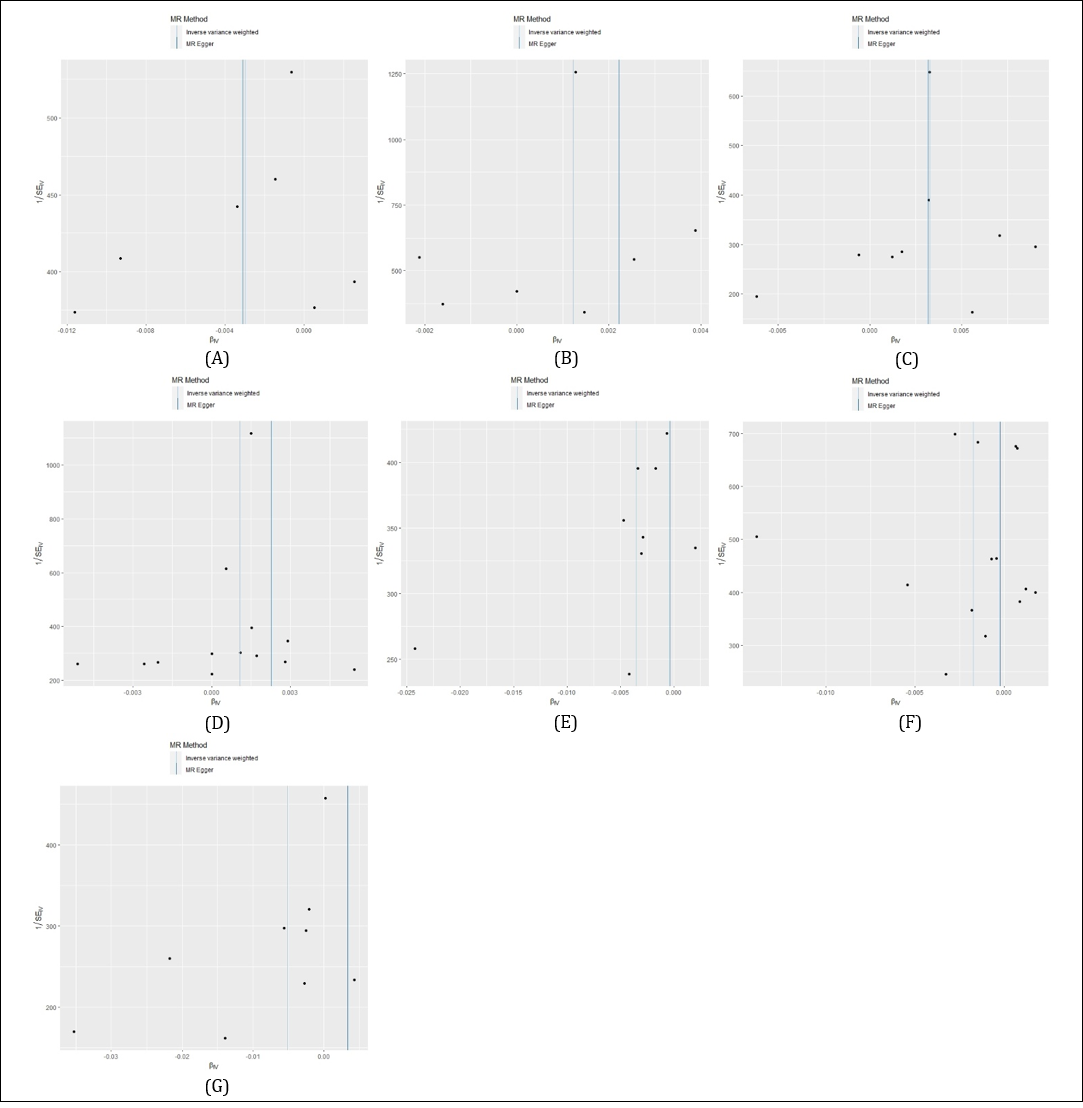


**eFigure 6**. Funnel of systemic inflammatory regulators-associated SNPs with estimated glomerular filtration rate. The genetic relationship between NGFβ, IL2ra, IL4, PDGF, SCF, SCGFβ, SDF1α and estimated glomerular filtration rate, denoted as A to G, respectively. Abbreviations: SCGFβ, stem cell growth factor beta; NGFβ, beta-nerve growth factor; IL, interleukin; SDF1α, stromal-cell-derived factor 1 alpha; SCF, stem cell factor; PDGF, platelet-derived growth factor BB.


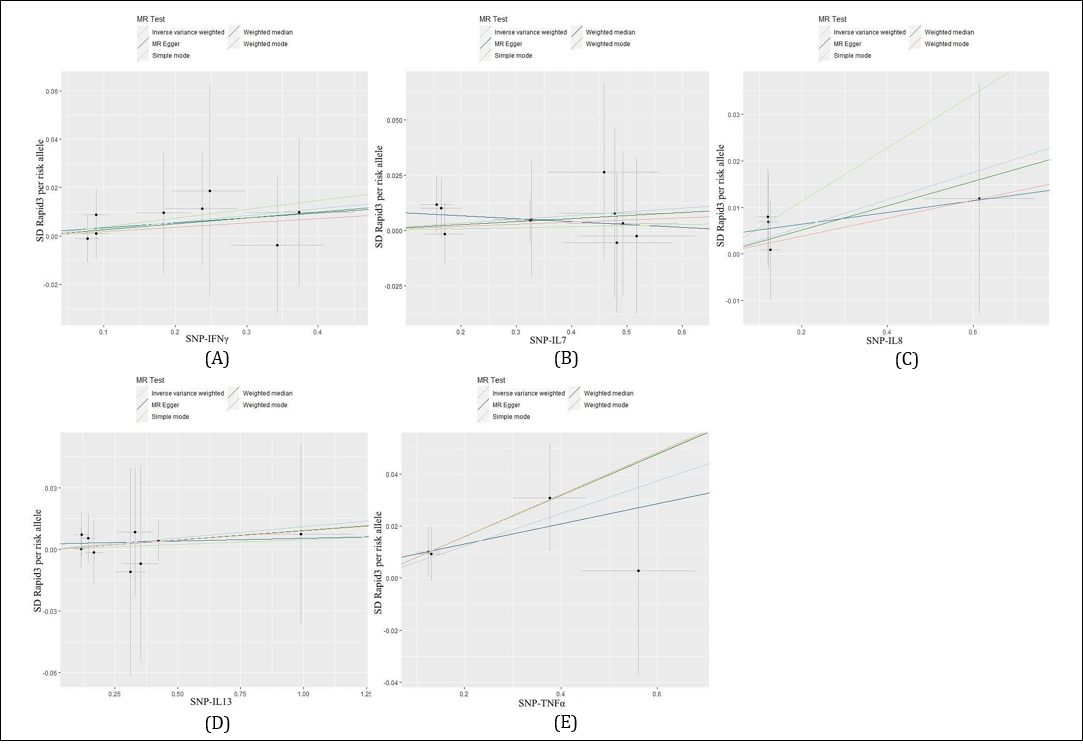


**eFigure 7**. Scatter plot of systemic inflammatory regulators-associated SNPs with risk of Rapid3. The genetic relationship between IFNγ, IL7, IL8, IL13, TNFα and rapid3, denoted as A to E, respectively. Abbreviations: IFNγ, interferon gamma; TNFα, tumor necrosis factor alpha; IL, interleukin; SD, standard deviation; Rapid3, rapid decline in renal function, i.e., a decline in eGFR of more than 3 mL/min/1.73 m^2^ per year.


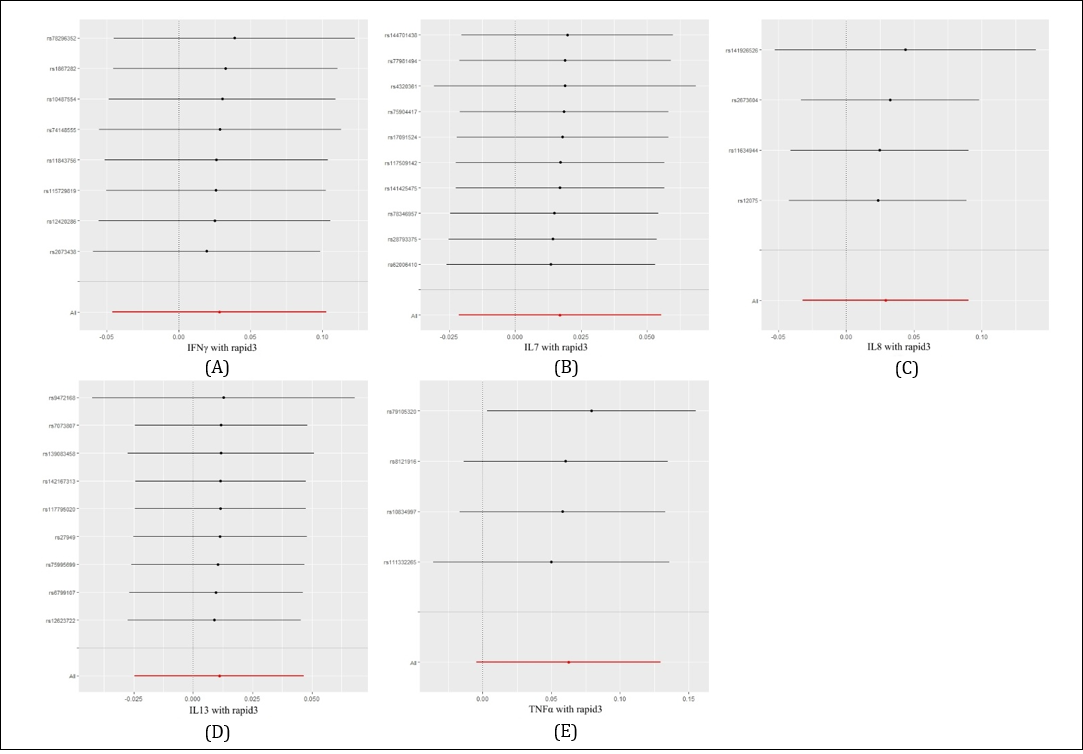


**eFigure 8**. MR leave-one-out sensitivity analysis of systemic inflammatory regulators-associated SNPs with risk of Rapid3. The genetic relationship between IFNγ, IL7, IL8, IL13, TNFα and rapid3, denoted as A to E, respectively. Abbreviations: IFNγ, interferon gamma; TNFα, tumor necrosis factor alpha; IL, interleukin; Rapid3, rapid decline in renal function, i.e., a decline in eGFR of more than 3 mL/min/1.73 m^2^ per year.


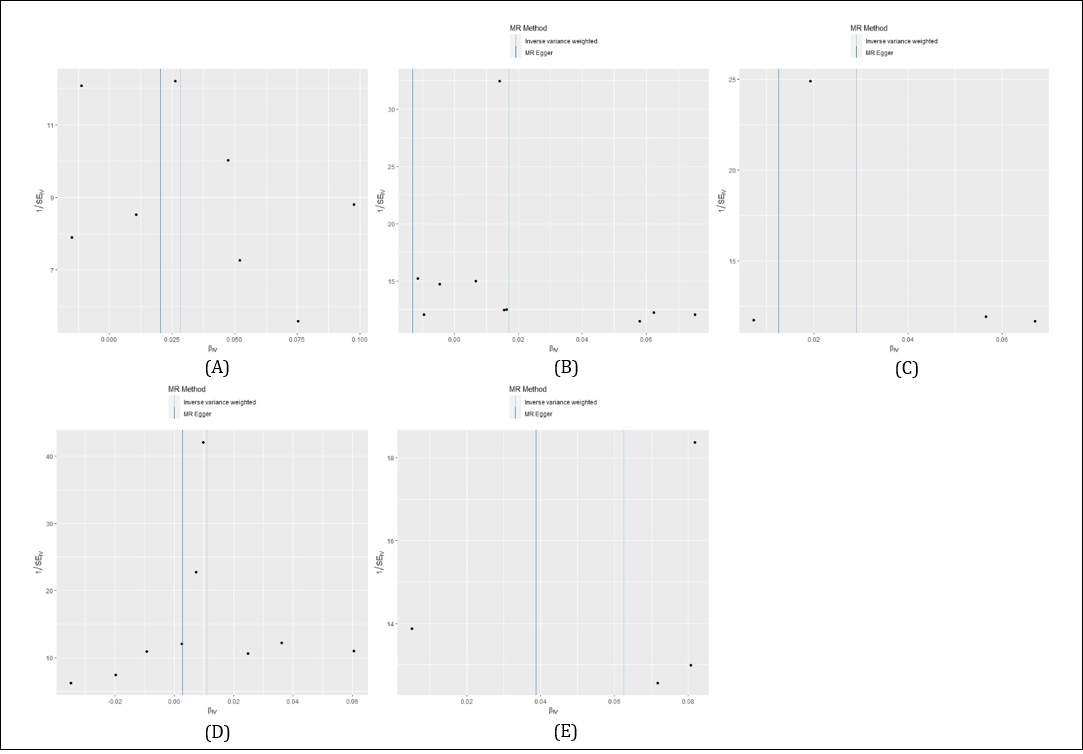


**eFigure 9**. Funnel of systemic inflammatory regulators-associated SNPs with risk of Rapid3. The genetic relationship between IFNγ, IL7, IL8, IL13, TNFα and rapid3, denoted as A to E, respectively. Abbreviations: IFNγ, interferon gamma; TNFα, tumor necrosis factor alpha; IL, interleukin; Rapid3, rapid decline in renal function, i.e., a decline in eGFR of more than 3 mL/min/1.73 m^2^ per year.


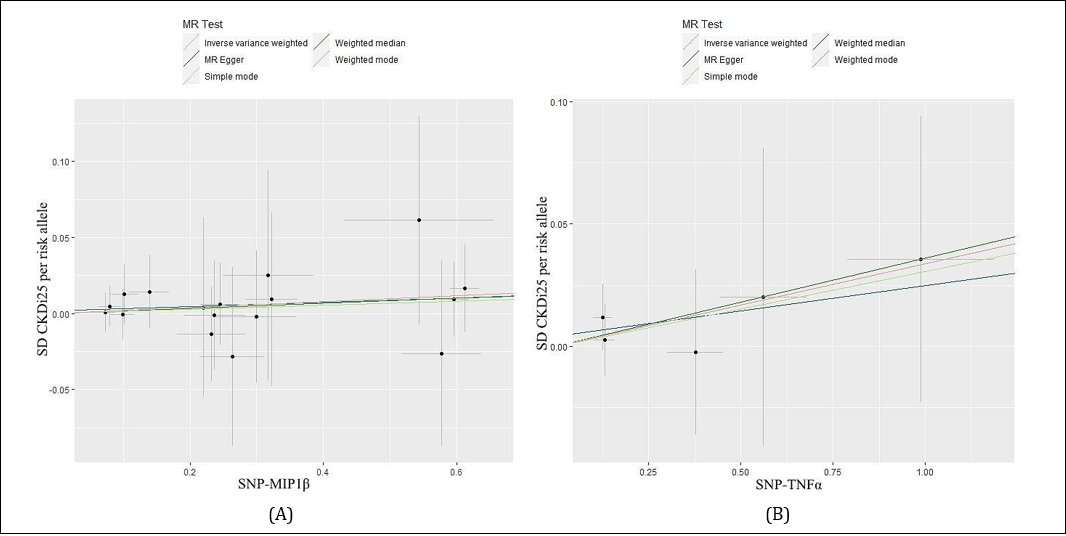


**eFigure 10**. Scatter plot of systemic inflammatory regulators-associated SNPs with risk of CKDi25. The genetic relationship between MIP1β, TNFα and CKDi25, denoted as A and B, respectively. Abbreviations: TNFα, tumor necrosis factor alpha; MIP1β, macrophage inflammatory protein 1β; SD, standard deviation; CKDi25, rapid progression to chronic kidney disease (CKD), i.e., a decrease in eGFR ≥ 25% of baseline, along with progression from no CKD to CKD.


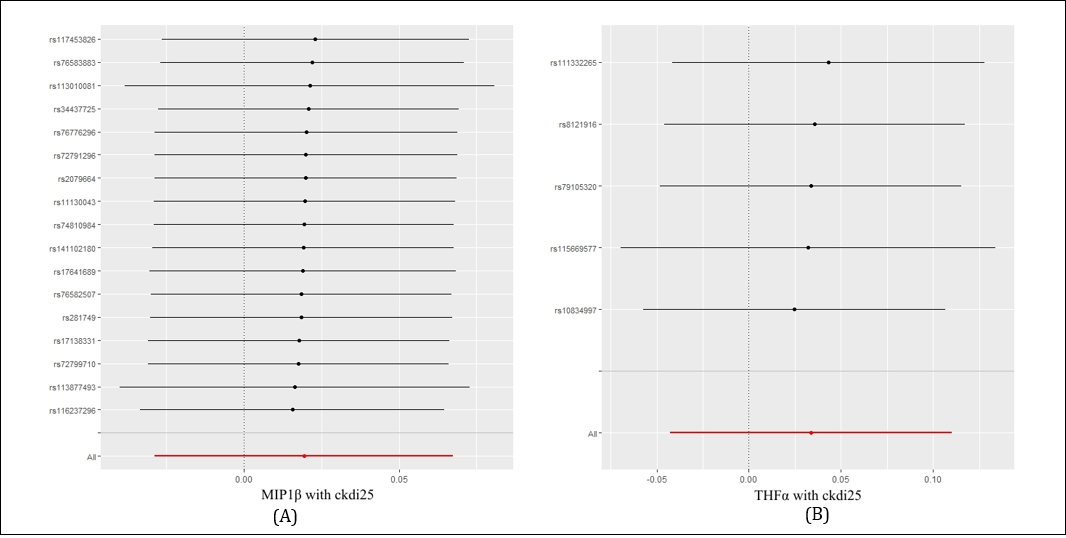


**eFigure 11**. MR leave-one-out sensitivity analysis of systemic inflammatory regulators-associated SNPs with risk of CKDi25. The genetic relationship between MIP1β, TNFα and CKDi25, denoted as A and B, respectively. Abbreviations: TNFα, tumor necrosis factor alpha; MIP1β, macrophage inflammatory protein 1β; CKDi25, rapid progression to chronic kidney disease (CKD), i.e., a decrease in eGFR ≥ 25% of baseline, along with progression from no CKD to CKD.


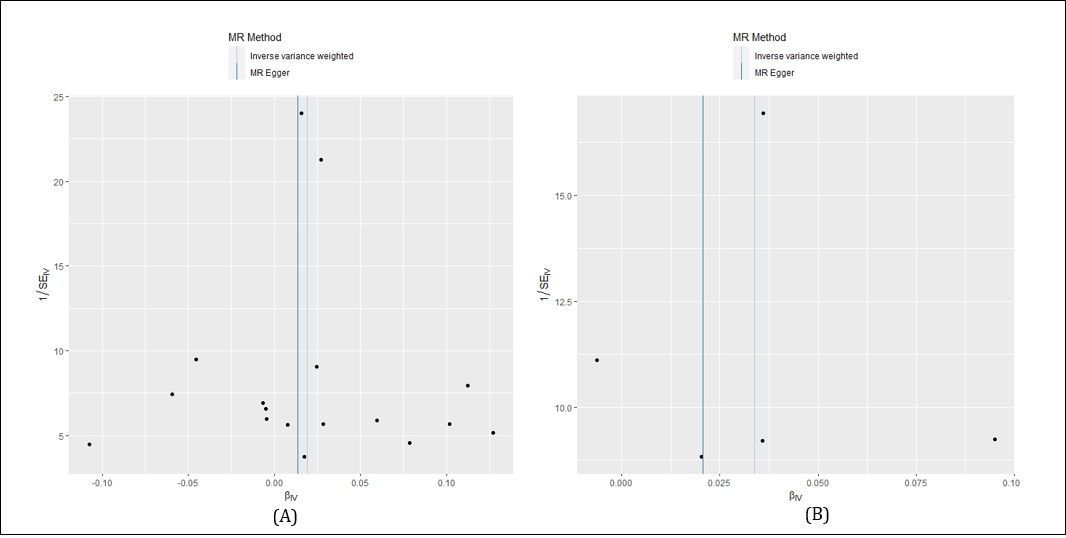


**eFigure 12**. Funnel of systemic inflammatory regulators-associated SNPs with risk of CKDi25. The genetic relationship between MIP1β, TNFα and CKDi25, denoted as A and B, respectively. Abbreviations: TNFα, tumor necrosis factor alpha; MIP1β, macrophage inflammatory protein 1β; SD, standard deviation; CKDi25, rapid progression to chronic kidney disease (CKD), i.e., a decrease in eGFR ≥ 25% of baseline, along with progression from no CKD to CKD.


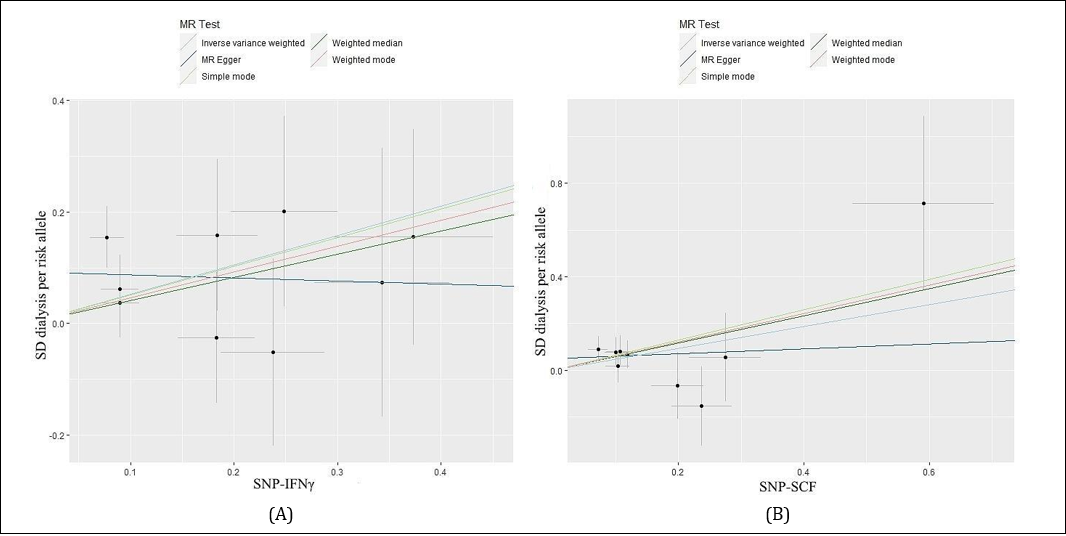


**eFigure 13**. Scatter plot of systemic inflammatory regulators-associated SNPs with risk of dialysis. The genetic relationship between IFNγ, SCF and dialysis, denoted as A and B, respectively. Abbreviations: IFNγ, interferon gamma; SCF, stem cell factor.

**
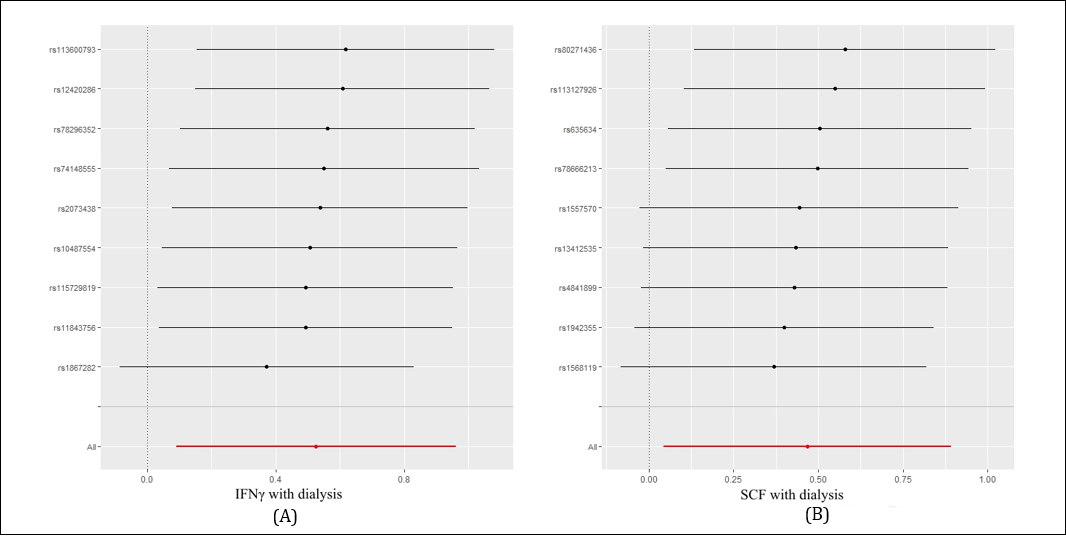
**

**eFigure 14**. MR leave-one-out sensitivity analysis of systemic inflammatory regulators-associated SNPs with risk of dialysis. The genetic relationship between IFNγ, SCF and dialysis, denoted as A and B, respectively. Abbreviations: IFNγ, interferon gamma; SCF, stem cell factor.

**
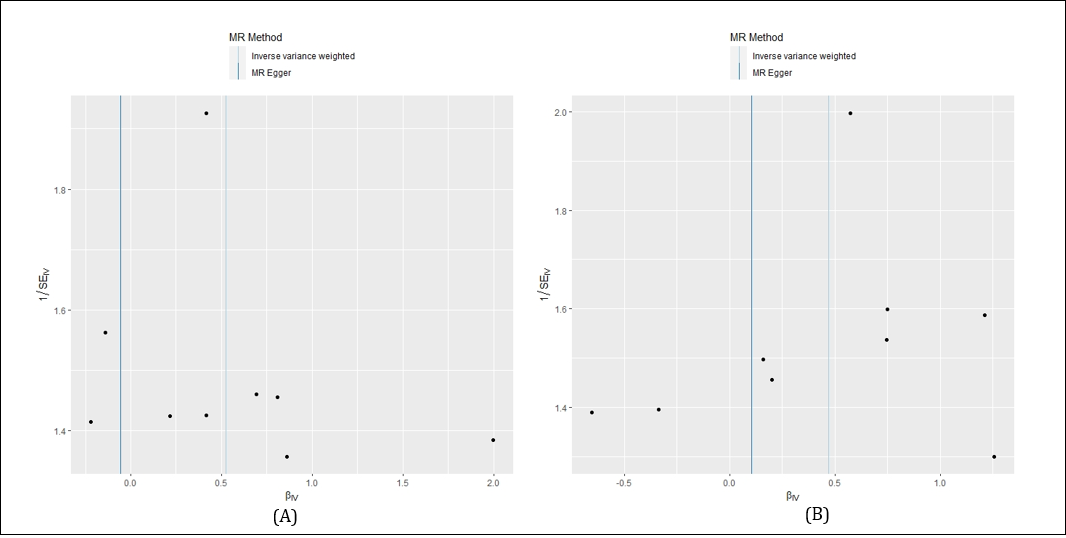
**

**eFigure 15**. Funnel of systemic inflammatory regulators-associated SNPs with risk of dialysis. The genetic relationship between IFNγ, SCF and dialysis, denoted as A and B, respectively. Abbreviations: IFNγ, interferon gamma; SCF, stem cell factor.

**eFigure 16.** Odds ratio for association of genetically predicted chronic kidney disease with systemic inflammatory regulators. CTACK, Cutaneous T-cell attracting; GROα, growth regulated oncogene alpha; SDF-1a, stromal-cell-derived factor 1 alpha; NGF-β, beta-nerve growth factor; SCGF-β, stem cell growth factor beta; IL, interleukin; TNF-β, tumor necrosis factor beta; TRAIL, TNF-related apoptosis inducing ligand; MR, mendelian randomization; CI, confidence internal; OR, odds ratio; IVW-FE, inverse-variance weighted fixed-effects MR; IVW-RE, inverse-variance weighted random-effects MR; WM, weighted median; IVR, instrumental variable ratio (Wald) estimator; SNP, single nucleotide polymorphism. P value for heterogeneity based on Cochran’s Q statistic for IVW, and Rücker’s Q for MR-Egger.

**eFigure 17.** Effect for association of genetically predicted estimated glomerular filtration rate with systemic inflammatory regulators. SCGF-β, stem cell growth factor beta; NGF-β, beta-nerve growth factor; IL, interleukin; SDF-1α, stromal-cell-derived factor 1 alpha; SCF, stem cell factor; PDGF, platelet-derived growth factor BB; MR, mendelian randomization; CI, confidence internal; OR, odds ratio; IVW-FE, inverse-variance weighted fixed-effects MR; IVW-RE, inverse-variance weighted random-effects MR; WM, weighted median; SNP, single nucleotide polymorphism. *P* value for heterogeneity based on Cochran’s Q statistic for IVW, and Rücker’s Q for MR-Egger.

**eFigure 18.** Odds ratio for association of genetically predicted Rapid3, CKDi25 and dialysis with systemic inflammatory regulators. IL, interleukin; TNF-α, tumor necrosis factor alpha; IFN-γ, interferon gamma; MIP1bβ, macrophage inflammatory protein 1 beta; SCF, stem cell factor; MR, mendelian randomization; CI, confidence internal; OR, odds ratio; IVW-RE, inverse-variance weighted random-effects MR; SNP, single nucleotide polymorphism; CKDi25, rapid progression to CKD, i.e., a decrease in eGFR ≥ 25% of baseline, along with progression from no CKD to CKD; Rapid3, rapid decline in renal function, i.e., a decline in eGFR of more than 3 mL/min/1.73 m^2^ per year. *P* value for heterogeneity based on Cochran’s Q statistic for IVW, and Rücker’s Q for MR-Egger.
